# Automated amplification-free digital RNA detection platform for rapid and sensitive SARS-CoV-2 diagnosis

**DOI:** 10.1038/s42003-022-03433-6

**Published:** 2022-05-26

**Authors:** Hajime Shinoda, Tatsuya Iida, Asami Makino, Mami Yoshimura, Junichiro Ishikawa, Jun Ando, Kazue Murai, Katsumi Sugiyama, Yukiko Muramoto, Masahiro Nakano, Kotaro Kiga, Longzhu Cui, Osamu Nureki, Hiroaki Takeuchi, Takeshi Noda, Hiroshi Nishimasu, Rikiya Watanabe

**Affiliations:** 1 Molecular Physiology Laboratory, Cluster for Pioneering Research, RIKEN Saitama, Japan; 2 CREST, Japan Science and Technology Agency, Saitama, Japan; 3 Department of Biological Sciences, Graduate School of Science, The University of Tokyo, Tokyo, Japan; 4 FUJIFILM Media Crest Co., Ltd, Tokyo, Japan; 5 Institute for Frontier Life and Medical Sciences, Kyoto University, Kyoto, Japan; 6 Division of Bacteriology, Department of Infection and Immunity, School of Medicine, Jichi Medical University, Tochigi, Japan; 7 Research Center for Drug and Vaccine Development, National Institute of Infectious Diseases, Tokyo, Japan; 8 Department of Molecular Virology, Tokyo Medical and Dental University, Tokyo, Japan; 9 Department of Chemistry and Biotechnology, Graduate School of Engineering, The University of Tokyo, Tokyo, Japan; 10 Research Center for Advanced Science and Technology, Structural Biology Division, The University of Tokyo, Tokyo, Japan; 11 Inamori Research Institute for Science, Kyoto, Japan

**Keywords:** Nanobiotechnology, Lab-on-a-chip, Single-molecule biophysics

## Abstract

In the ongoing COVID-19 pandemic, rapid and sensitive diagnosis of viral infection is a critical deterrent to the spread of SARS-CoV-2. To this end, we developed an automated amplification-free digital RNA detection platform using CRISPR-Cas13a and microchamber device (opn-SATORI), which automatically completes a detection process from sample mixing to RNA quantification in clinical specimens within ~9 min. Using the optimal Cas13a enzyme and magnetic beads technology, opn-SATORI detected SARS-CoV-2 genomic RNA with a LoD of < 6.5 aM (3.9 copies μL^−1^), comparable to RT-qPCR. Additionally, opn-SATORI discriminated between SARS-CoV-2 variants of concern, including alpha, delta, and omicron, with 98% accuracy. Thus, opn-SATORI can serve as a rapid and convenient diagnostic platform for identifying several types of viral infections.

## Abstract (web summary)

An automated amplification-free digital RNA detection platform using CRISPR-Cas13a and magnetic bead technology implemented in a microchamber device demonstrates sensitive SARS-CoV-2 genomic RNA detection and variant discrimination.

## Introduction

More than 2 years have passed since the spread of the novel coronavirus SARS-CoV-2 in December 2019, but the occurrence of infections has not abated. Additionally, a worldwide epidemic of the highly infectious omicron variant is currently prevalent^1^. From the standpoint of public health, early diagnosis and isolation of infected persons are effective in preventing the spread of infection. Rapid, sensitive, and frequent testing is important for early diagnosis^2^. For the diagnosis of SARS-CoV-2 infection, reverse transcription-quantitative polymerase chain reaction (RT-qPCR) has been used as the “gold standard” platform, due to its high sensitivity (~2–20 aM (~1–10 copies μL^−1^)) and quantitative output^3^. In addition, CRISPR-based nucleic acid detection methods, such as SHERLOCK and DETECTR, have also gained attention because of their high sensitivity (~2–20 aM (~1–10 copies μL^−1^)), relatively short assay time (several tens of minutes), and compatibility with compact detection platforms^4–10^. Most CRISPR-based methods involve isothermal amplification of target nucleic acids and the fluorescence or colorimetric readout of the amplified nucleic acids using the CRISPR-Cas enzymes, Cas12a or Cas13a. The amplification step is indispensable to increase detection sensitivity, but it prolongs the time to detection (at least several tens of minutes). Recently, we developed an amplification-free RNA detection platform called SATORI (CRISPR-based amplification-free digital RNA detection) by combining the CRISPR-based nucleic acid detection and microchamber technologies^11^. SATORI can detect single-stranded RNA (ssRNA) such as SARS-CoV-2 RNA, in 5 min, with an analytical limit of detection (LoD) of ~5 fM (~3 × 10^3^ copies μL^−1^), enabling rapid diagnosis of SARS-CoV-2 RNA infection in most clinical specimens from patients (~10^3^–10^6^ copies μL^−1^ on average)^12,13^. However, the LoD should be <10^2^ copies μL^−1^ for highly effective screening^2^. In addition, SATORI contains several manual handling steps, which can cause human errors and reduce assay accuracy. To resolve these issues, we developed a fully automated version of SATORI, termed as automated platform on SATORI (opn-SATORI). We demonstrated that opn-SATORI can detect SARS-CoV-2 RNA in clinical specimens with high sensitivity comparable to that of RT-qPCR, and discriminate between SARS-CoV-2 variants in ~9 min with an accuracy of 98%.

## Results

### Development of automated platform

**Fig. 1:**
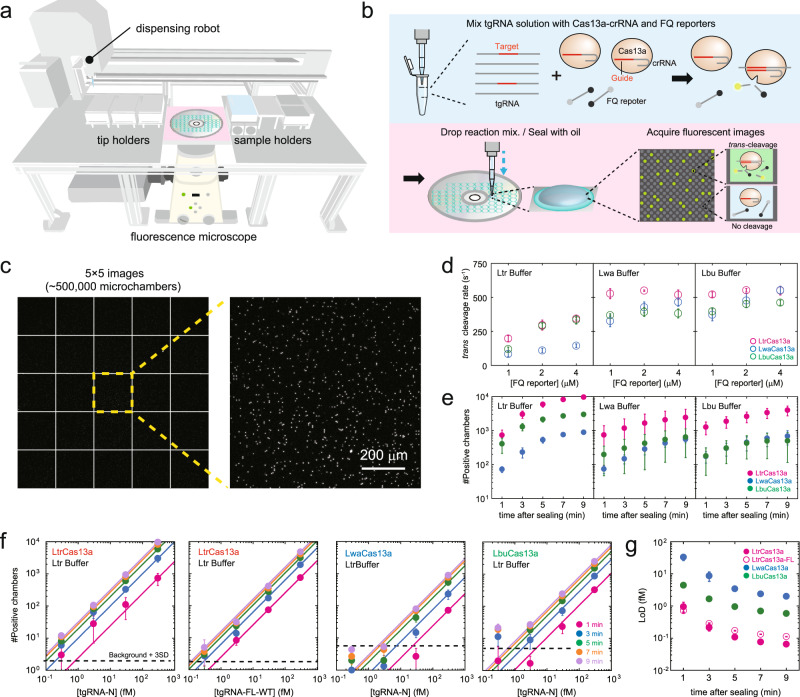
Automated platform for amplification-free digital RNA detection with a CRISPR-Cas13a and CD-based microchamber device. **a** Schematic illustration of the automated platform consisting of a fluorescence microscope and a custom-made dispensing robot. **b** Assay procedures. Upon tgRNA binding, the Cas13a-crRNA complex cleaves FQ reporters, resulting in increased fluorescence in the microchamber. **c** Representative fluorescence images obtained with 300 fM tgRNA. Using tiling-based imaging, 25 fluorescence images containing ~500,000 microchambers can be acquired in less than 2 min. Scale bar is 200 μm. **d** Comparison of the *trans*-cleavage activities of the Cas13a orthologs at the single-molecule level using different concentrations of FQ reporters. **e** Comparison of the number of positive chambers obtained with the different Cas13a orthologs. The Ltr-N2/Lwa-N2/Lbu-N2 and the SARS-CoV-2 N-gene (300 fM) were used as the crRNA and tgRNA, respectively. **f** The number of positive chambers obtained with the different Cas13a orthologs at different concentrations of the N-gene or the whole genome RNA of SARS-CoV-2. The data were acquired at 1, 3, 5, 7, and 9 min after oil sealing. The solid lines indicate linear regressions. The values of the background mean +3 S.D. are indicated by dotted lines. **g** Time courses of LoD values determined from (**f**) (*n* = 3 technical replicates for **d**–**g**).

To automate SATORI assays, we developed an RNA detection platform consisting of three main components: a compact disk (CD) device, a fluorescence microscope, and a dispensing robot (Fig. 1a, Supplementary Figs. 1, 2a). The CD-based device contains ~10^8^ cylindrical femtoliter microchambers (V = ~30 fL, ϕ = 3.5 μm, h = 3.5 μm), which are compartmentalized by ring-shaped, acrylic-resin enclosures (Supplementary Fig. 1). Unlike SATORI^11^ and other digital bioassays^14,15^, our CD-based device does not require complicated solution-exchange processes in a microfluidic channel, and can complete processes from sample mixing to image analysis in a fully automated manner (Fig. 1b, Supplementary Fig. 2b). The developed platform automatically enables the following detection process: (i) preparation of the reaction solution by mixing the enzyme solution containing pre-assembled Cas13a-crRNA complexes and fluorophore quencher-labeled ssRNA reporters (FQ reporter) with sample solution containing the crRNA-complementary ssRNA target (tgRNA) in a test-tube, (ii) dropping of the reaction solution into microchambers on the CD device, (iii) sealing of the microchambers with a drop of oil, (iv) accumulation of fluorescent products derived from Cas13a-mediated FQ-reporter cleavage (*trans*-cleavage), (v) acquisition of images from ~500,000 chambers using a fluorescence microscope, and (vi) counting the number of fluorescent (positive) chambers to quantify tgRNA copies in the sample (Fig. 1c, Supplementary Fig. 2b). Of note, at low concentrations of the tgRNA, each chamber stochastically contains one or zero Cas13a-crRNA-tgRNA complex according to a Poisson distribution as previously reported^11^. Thus, the tgRNA copies can be quantified from the number of the positive chambers^11,15,16^. The entire process can be repeated automatically, and 48 samples can be analyzed using a single setup.

### Screening of Cas13a orthologs

To improve detection sensitivity, we screened Cas13a orthologs and optimized the buffer conditions. Cas13a from *Leptotrichia wadei* (LwaCas13a) and *Leptotrichia buccalis* (LbuCas13a) have been used for CRISPR-based nucleic acid detection, because of their robust *trans*-cleavage activity^5,17^. In addition, a recent study showed that Cas13a from *Leptotrichia trevisanii* (LtrCas13a) shares sequence identity with LwaCas13a (88%) and LbuCas13a (83%) (Supplementary Fig. 3), and inhibited bacterial growth comparably to LwaCas13a^18^. Thus, we compared Cas13a orthologs at the single-molecule level with our RNA detection platform, using two crRNA guides (crRNA-N1 and crRNA-N2, Supplementary Table 1) and a SARS-CoV-2 N-gene RNA, as shown in our previous SATORI assays^11^. LtrCas13a showed robust and rapid *trans-*cleavage activity (>250 s^−1^ at 2 μM FQ reporter) and resulted in the most rapid increase in fluorescence intensity under different buffer conditions, with the fluorescence signals reaching a plateau in 2 min at >2 μM FQ reporter (Fig. 1d, Supplementary Figs. 4, 5, Supplementary Table 2). Notably, the three Cas13a enzymes followed the reasonable Michaelis–Menten kinetics^19^ (Supplementary Fig. 5h), demonstrating the validity of the kinetic evaluation. Furthermore, the use of LtrCas13a resulted in more positive chambers, as compared to LwaCas13a and LbuCas13a, under all three buffer conditions, including the previously reported buffers for LwaCas13a (Lwa buffer) and LbuCas13a (Lbu buffer) (Fig. 1e, Supplementary Figs. 4, 6). Using LtrCas13a, we observed the largest number of positive chambers in a new reaction buffer (Ltr buffer), i.e., more LtrCas13a molecules were activated upon tgRNA binding in Ltr buffer. The number of positive chambers in Ltr buffer increased linearly, depending on the tgRNA concentration (300 aM–300 fM) (Fig. 1f, Supplementary Fig. 6c). At ~7 min after the start of the assay (3 min after oil sealing), LtrCas13a detected the N-gene RNA and the whole genome RNA from SARS-CoV-2, with LoD values of 220 and 280 aM (1.3 × 10^2^ and 1.7 × 10^2^ copies μL^−1^) (“Materials and methods”) (Fig. 1g, Supplementary Fig. 6d), which are ~37- and ~8-fold lower than those of LwaCas13a (8.1 fM (4.9 × 10^3^ copies μL^−1^) and LbuCas13a (1.7 fM (1.0 × 10^3^ copies μL^−1^)), respectively, using our platform. The LoD values were improved by prolonged incubation (~10-fold improvement in 10 min after oil sealing), because the number of positive chambers were increased likely due to an increase in the number of activated Cas13 molecules. Given the rapid, sensitive, and robust *trans*-cleavage activity obtained, we concluded that the combination of LtrCas13a and Ltr Buffer is optimal for use with our RNA detection platform.

### Improvement of detection sensitivity of viral RNA

**Fig. 2:**
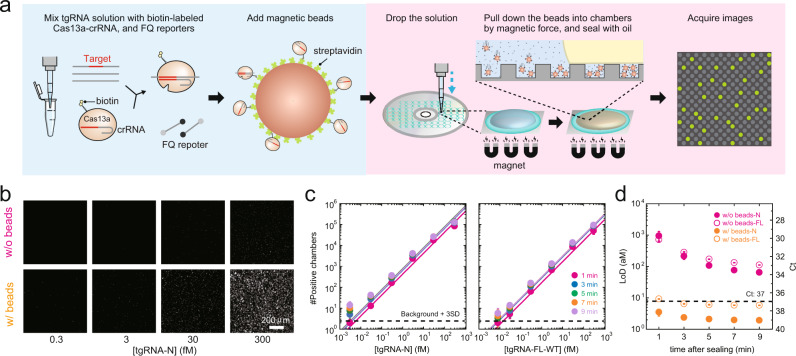
opn-SATORI. **a** Schematic illustration of opn-SATORI. Biotin-labeled LtrCas13a-crRNA-tgRNA complexes were attached to streptavidin-coated magnetic beads and enriched in microchambers by a magnetic force before the sealing with oil. **b** Representative images obtained with or without magnetic bead treatment at different concentrations of the SARS-CoV-2 N-gene. **c** The number of positive chambers obtained using different concentrations of SARS-CoV-2 N-gene or whole genome RNA. The solid lines indicate linear regressions. The values of the background mean + 3 S.D. are indicated by dotted lines. **d** Time courses of LoD values with or without the magnetic bead treatment, for detection of the SARS-CoV-2 N-gene or whole genome RNA. The dotted line indicates the Ct value of 37 for the SARS-CoV-2 whole genome RNA evaluated by RT-qPCR. (*n* = 3 technical replicates for **c** and **d**).

Because our platform detects tgRNA molecules that are captured stochastically in the microchamber arrays with a total volume of ~15 nL (500,000 × ~30 fL), >99% of the tgRNA molecules are discarded in the oil sealing process, thereby resulting in reduced sensitivity. To overcome this drawback and further improve the sensitivity, we used biotin-streptavidin magnetic-bead technology to enrich tgRNA molecules inside the microchambers. Streptavidin-coated magnetic beads were added to the reaction solution containing biotin-labeled LtrCas13a-crRNA complexes, tgRNAs, and the FQ reporters to capture LtrCas13a-crRNA-tgRNA complexes on the beads, and subsequently, the mixture was dropped onto the CD device, the complexes were concentrated into the chambers by magnetic force, and the chambers were sealed with oil (Fig. 2a). Notably, the procedure was automatically completed within 5 min (Supplementary Fig. 7) and resulted in a ~50-fold increase in the number of positive chambers as compared to that without the magnetic bead treatment (Fig. 2b), indicating the successful enrichment of LtrCas13a-crRNA-tgRNA complexes in the microchambers. The number of positive chambers increased linearly with a wide range of tgRNA concentrations (8 aM–30 fM) (Fig. 2c), confirming that this platform can be used for tgRNA quantification. At ~9 min after the start of the assay (3 min after oil sealing), the platform detected the N-gene RNA and the whole genome RNA of SARS-CoV-2, with LoD values of 2.4 and 6.5 aM (1.4 and 3.9 copies μL^−1^), respectively (Fig. 2d). The number of positive chambers and the LoD values remained nearly constant after oil sealing, indicating that enrichment of the LtrCas13a-crRNA-tgRNA complexes accelerated the Cas13a activation. We termed our automated RNA detection platform coupled with biotin-streptavidin magnetic beads as an automated platform on SATORI (opn-SATORI).

### Discrimination of SARS-CoV-2 variants of concern

**Fig. 3:**
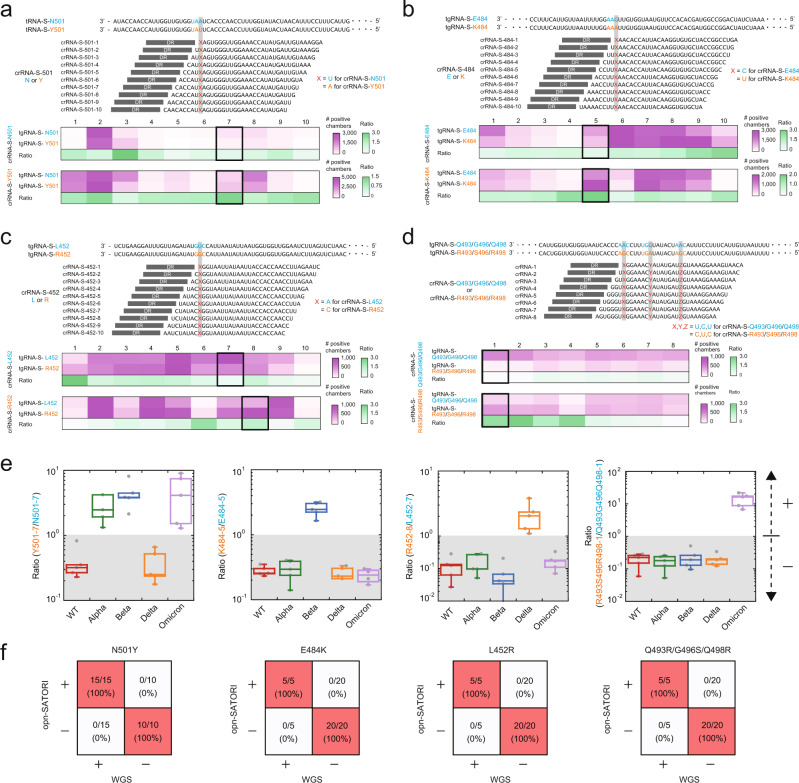
Discrimination of the S-gene mutations of SARS-CoV-2 variants. **a**–**d** Design and screening of crRNA for detection of the N501Y (**a**), E484K (**b**), L452R (**c**), and Q493R/G496S/Q498R (**d**) mutations in the SARS-CoV-2 S-gene (*n* = 3 technical replicates). **e** Discrimination of S-gene mutations in the SARS-CoV-2 variants using opn-SATORI (*n* = 5 technical replicates). Wuhan-Hu1, B.1.1.7, B.1.351, B.1.617, and B.1.1.529 were used as WT, α, β, δ, and ο variants, respectively. The definition of having a mutation is that the ratio value is greater than 1.0. In the box plots, the boundary of the box closest to zero indicates the 25th percentile, and black line within the box marks the median, and the boundary of the box farthest from zero indicates the 75th percentile. The error bars indicate S.D. **f** Detection specificity of opn-SATORI. All data were obtained at 3 min after oil sealing.

We sought to distinguish between the SARS-CoV-2 variants using opn-SATORI (Fig. 3). As a proof-of-concept experiment, we targeted the N501Y, E484K, and L452R spike mutations in the corresponding variants of concern, i.e., B.1.1.7 (*α*), B.1.351 (*β*), and B.1.617 (*δ*)^20^. For each mutation, we designed crRNAs with a single mismatch in spacer positions 1–10 relative to either wild-type (WT) or mutant SARS-CoV-2 RNA (thus, 20 crRNAs were designed for each mutation), and then evaluated the number of positive chambers using 120-nt WT or mutant S-gene RNAs (Fig. 3a–c, Supplementary Tables 3–5). Among the crRNAs tested, six crRNAs (N501-7, Y501-7, E484-5, K484-5, L452-7, and R452-8) resulted in a ~2-fold decrease in the number of positive chambers when activated by mismatched tgRNA, although the number of positive chambers varied regardless of the mismatch position, as observed in previous studies^4^ (Fig. 3a–c). Using the three crRNA pairs (N501-7/Y501-7, E484-5/K484-5, and L452-7/R452-8), we used opn-SATORI to evaluate the ratio of the number of positive chambers to the whole genome RNAs from WT SARS-CoV-2 and the α (N501Y), β (N501Y/E484K), and δ (L452R) variants. Notably, we observed significant differences in the ratios between WT SARS-CoV-2 and each variant in ~9 min after the start of the assay (3 min after oil sealing), thereby enabling the rapid and precise discrimination of the SARS-CoV-2 variants (Fig. 3e, f, Supplementary Figs. 8, 9). Furthermore, the use of opn-SATORI with a crRNA pair (Q493G496Q498-1 and R493S496R498-1) discriminated the B.1.1.529 (ο) variant (Fig. 3d–f, Supplementary Figs. 8, 9). Together, these results demonstrate that the use of opn-SATORI with appropriate crRNA pairs targeting a specific mutation can detect emerging SARS-CoV-2 variants.

### Clinical validation

**Fig. 4:**
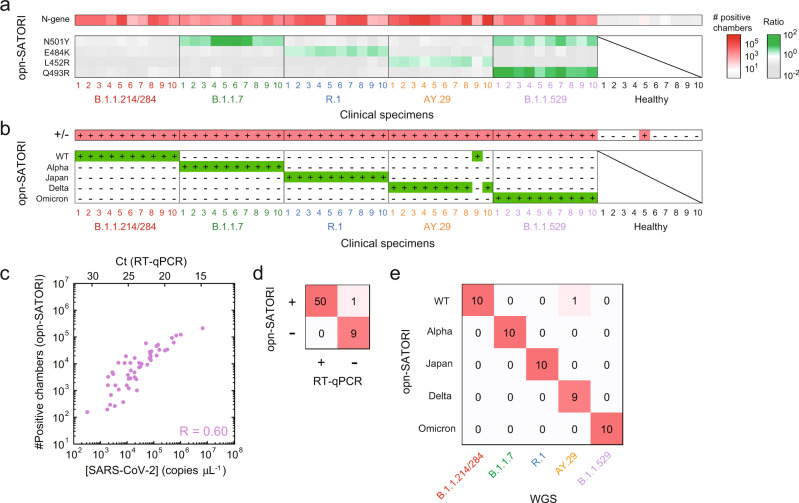
Clinical validation. **a**, **b** Detection of SARS-CoV-2 variants, and discrimination of the S-gene mutations in clinical specimens. B.1.1.214/284, B.1.1.7, R.1, AY.29, and B1.1.529 were WT, alpha, Japan, delta, and omicron variants, respectively. The definition of having a mutation is that the ratio value is greater than 1.0. **c** Comparison of opn-SATORI and RT-qPCR for quantification of the SARS-CoV-2 genomic RNA copy number. **d** Comparison of opn-SATORI and RT-qPCR results in SARS-CoV-2 detection. **e** Comparison of opn-SATORI and WGS results in variant discrimination.

We examined whether opn-SATORI could be used for the diagnosis of SARS-CoV-2 infection (Fig. 4). Using the crRNA targeting the N-gene RNA, we evaluated the number of positive chambers from 50 human nasopharyngeal swab-derived RNA samples of patients with Ct values of 15–30 (10 WT, 10 alpha, 10 Japan, 10 delta, and 10 omicron variants) and 10 RNA samples from healthy persons (Supplementary Fig. 10, Supplementary Table 6). The number of positive chambers for the samples correlated well with their copy numbers (Ct values) determined by RT-qPCR, with a correlation coefficient of 0.60 (Fig. 4c). In addition, Bland–Altman analysis showed good agreement between the results obtained by opn-SATORI and RT-qPCR (Supplementary Fig. 11), demonstrating the ability of opn-SATORI to quantify the copy number of SARS-CoV-2 RNA in the sample. Accordingly, we distinguished between SARS-CoV-2 positive and negative samples, with the accuracy of 98% and the positive precision rate of 100% (Fig. 4a, d), highlighting the robustness of opn-SATORI for clinical diagnosis. Furthermore, we sought to conduct the variant discrimination by detecting the N501Y, E484K, L452R, and Q493R/G496S/Q498R spike mutations, which were confirmed by whole genome sequencing (WGS) (Supplementary Table 6). Using the four crRNA pairs (N501-7/Y501-7, E484-5/K484-5, L452-7/R452-8, and Q493G496Q498-1/R493S496R498-1), we detected the N501Y, E484K, L452R, and Q493R/G496S/Q498R mutations in the aforementioned clinical samples, with 100%, 100%, 90%, and 100% concordance with the WGS results, respectively (Fig. 4a, Supplementary Fig. 12), thereby enabling the discrimination of the SARS-CoV-2 variants of concern with the accuracy of 98% (Fig. 4b, e). Together, these results demonstrate the potential of opn-SATORI for rapid and precise diagnosis of SARS-CoV-2 infection.

## Discussion

Here, we demonstrated that opn-SATORI is a sensitive, rapid, and automated platform for ssRNA detection that can precisely diagnose SARS-CoV-2 and its variants. We performed detailed biochemical characterization of the enzyme kinetics (*trans*-cleavage activity) of several Cas13a orthologs and identified LtrCas13a as the optimal enzyme for our platform, suggesting that LtrCas13a may help improve the sensitivity and assay time of other bulk CRISPR-based methods, such as SHERLOCK^4^. Of note, the *trans-*cleavage activity of the Cas13a enzymes observed in this study was comparable to those determined by other single-molecule assays^21^, but higher than those determined by bulk assays^22^. One possible explanation for the differences is that single-molecule assays can identify and measure cleavage by active Cas13-crRNA-tgRNA complex molecules only, while bulk assays measure the averaged cleavage by active and potential inactive complexes^23^.

We integrated biotin-streptavidin magnetic-bead technology into our platform, and substantially improved the detection sensitivity of opn-SATORI. The sensitivity obtained (LoD of 2.4 aM (1.4 copies μL^−1^)) was >1000-fold higher than the previously reported SATORI platform (~5 fM (~3 × 10^3^ copies μL^−1^)), and is >1000-times higher than antigen tests (~17 fM (1.0 × 10^4^ copies μL^−1^))^24^, and is comparable with RT-qPCR (~2–20 aM (~1–10 copies μL^−1^))^3^ and other CRISPR-based methods that use a pre-amplification step, such as SHERLOCK and DETECTR (2–20 aM (~1–10 copies μL^−1^)). Importantly, opn-SATORI detected SARS-CoV-2 RNA without amplification in ~9 min (time from sample mixing to image analysis), whereas RT-qPCR and other CRISPR-based methods take at least several tens of minutes for detection. We previously demonstrated that SATORI is not substantially affected by contaminants from human swab^11^, suggesting that opn-SATORI may function more rapidly and simply by incorporation of nucleic acid extraction-free procedures used in RT-qPCR and CRISPR-based methods^10,25^. Notably, the use of opn-SATORI with appropriate crRNA pairs can identify single genetic mutations and distinguish SARS-CoV-2 variants with high specificity (98%). Within a few days, we completed all the processes from the design of crRNAs targeting the spike mutations in the B.1.1.529 (ο) variant to successful discrimination of the variant, highlighting the utility of opn-SATORI for the effective screening of emerging SARS-CoV-2 variants.

We envision that the use of opn-SATORI can be implemented in society and be widely used in town clinics, airport quarantine stations, and other places. The cost of device fabrication for opn-SATORI (conventional CD-based microchamber devices) is ~US$0.01 per assay, lower than that of SATORI (US$5.20 per assay)^11^. The cost of the assay reagents for opn-SATORI is ~US$2 per assay (Supplementary Table 7), and is comparable with those for RT-qPCR and antigen tests (US$1.21–4.39 per assay)^25^. Because the opn-SATORI platform uses a dispensing robot and a fluorescence microscope and is relatively large (approximately 1 m^2^) (Supplementary Fig. 2a), it is important to develop a more compact platform for common use. Given that CRISPR-Cas13a-based methods have been used for miRNA detection in cancer diagnosis^26^, opn-SATORI may serve as a versatile platform for diverse applications, including diagnosis of viral infections and evaluation of disease-related biomarkers for liquid biopsy. We believe that opn-SATORI will be a key technology for high-throughput genetic diagnostics.

## Methods

### Fabrication of CD-based microchamber devices

The original mold was prepared via photolithography using a 3D drawing system (DWL66 + , Hidelberg Instruments), and the Ni replica was fabricated by electrocasting. CD-based microchamber devices were fabricated using polycarbonate resin with a molding machine using the mold described above (SD40ER, Sumitomo Heavy Industries). A UV-curable acrylic resin (5X649H, CHEMITECH INC.) and a dispensing robot (SHOTmini 200SX SM200S, Musashi Engineering) were used to fabricate a ring-shaped enclosure with a 7 mm diameter on the CD.

### Preparation of the Cas13a proteins

For expression of LtrCas13a and LwaCas13a, *Escherichia coli* Rosetta 2 (DE3) was transformed with the pET-LtrCas13a or pET-LwaCas13a plasmids, and the cells were cultured in 2.5 L of LB medium containing kanamycin. When the OD_600_ values reached 0.6–1.0, the cells were cooled on ice for 10 min, and were further cultured at 20 °C for 20 h with 0.1 mM IPTG. Bacterial cells were collected by centrifugation, suspended in 40 mL buffer A (20 mM Tris-HCl (pH 8.0), 1 M NaCl, 20 mM imidazole, 3 mM β-mercaptoethanol and 1 mM phenylmethylsulfonyl fluoride), and disrupted by sonication (Q500, QSONICA). After centrifugation at 15,000 rpm for 20 min, the supernatant was incubated with Ni-NTA agarose (Qiagen) at 4 °C for 1 h. The mixture was subsequently transferred to an Econo column (Bio-Rad). The resin was washed with buffer B (20 mM Tris-HCl (pH 8.0), 0.3 M NaCl, 20 mM imidazole and 3 mM β-mercaptoethanol), and the protein was eluted with buffer C (20 mM Tris-HCl (pH 8.0), 0.3 M NaCl, 300 mM imidazole and 3 mM β-mercaptoethanol). The protein was then loaded onto a HiTrap SP HP column (Cytiva) equilibrated with buffer D (50 mM HEPES-KOH (pH 7.5), 0.3 M NaCl and 0.5 mM Tris(2-carboxyethyl)phosphine (TCEP)). The protein was eluted using a linear gradient from 0.3 to 2.0 M NaCl over 7 column volumes. The protein was further purified by size exclusion chromatography (Enrich SEC 650, Bio-Rad) with buffer E (50 mM HEPES-KOH (pH 7.5), 0.5 M NaCl, and 0.5 mM TCEP).

For expression of LbuCas13a, *E. coli* Rosetta 2 (DE3) was transformed with the p2CT-MBP-LbuCas13a plasmid, and the cells were cultured in 2 L of LB medium containing ampicillin. When the OD_600_ values reached 0.6–0.8, the cells were cooled on ice for 10 min and cultured at 20 °C for 20 h with 0.1 mM IPTG. Bacterial cells were collected by centrifugation, and suspended in 30 mL buffer A, and disrupted by sonication. After centrifugation at 15,000 rpm for 20 min, the supernatant was incubated with Ni-NTA agarose at 4 °C for 1 h. The mixture was transferred to an Econo column, and the protein was eluted with buffer C after washing with buffer B. TEV proteases were added to the protein at 1/50th the molar amount of the protein, and the mixture was dialyzed overnight at 4 °C against buffer F (20 mM Tris-HCl (pH 8.0), 0.3 M NaCl, 40 mM imidazole and 3 mM β-mercaptoethanol). The dialyzed sample was loaded onto a Ni-Sepharose High Performance column (GE Healthcare) equilibrated with buffer B, and the flowthrough was collected. The purified protein was loaded onto a HiTrap Heparin HP column (1 mL; GE Healthcare), equilibrated with buffer D, and eluted using a linear gradient from 0.3 to 2.0 M NaCl. The protein was further purified via size exclusion chromatography (Superdex 200 Increase, Cytiva) equilibrated with buffer E.

### Preparation of the guide and target RNAs

Some crRNAs were purchased from GeneDesign Inc. (Supplementary Table 1). The other crRNAs and tgRNAs were prepared via in vitro transcription using T7 RNA polymerase and a partially double-stranded DNA template (Supplementary Table 5) in buffer containing 50 mM Tris-HCl (pH 8.0), 40 mM KCl, 20 mM MgCl_2_, 5 mM DTT, 2 mM spermidine, 5 mM ATP, 5 mM UTP, 5 mM GTP, 5 mM CTP, 20 mM GMP and pyrophosphatase (SIGMA) at 37 °C for 1 h. To remove double-stranded RNA contaminants^27^, the mixture was incubated with RNaseIII (New England Biolabs) at 37 °C for 30 min, and the RNA was purified using RNeasy mini kit (Qiagen) and 8% native polyacrylamide gel electrophoresis^11^. The RNA product was extracted from the polyacrylamide gel into nuclease-free water. RNA concentrations were determined from the A_260_ value that was measured using a NanoDrop spectrophotometer.

### Preparation of the SARS-CoV-2 genomic RNAs

Isolated SARS-CoV-2 strains, SARS-CoV-2/Hu/DP/Kng/19-027 (Wuhan lineage), hCoV-19/Japan/QHN002/2020 (B.1.1.7 lineage), hCoV-19/Japan/TY8-612/2021 (B.1.351 lineage), hCoV-19/Japan/TY11-927-P1/2021 (B1.617.2 lineage), and CoV-19/Japan/TY38-873P0/2021 (B.1.1.529 lineage) were propagated in VeroE6/TMPRSS2 cells (JCRB 1819), which were cultured in Dulbecco’s modified Eagle’s medium (DMEM, Sigma-Aldrich) containing 5% fetal calf serum (FCS) and 1% penicillin/streptomycin at 37 °C with 5% CO_2_. Viral supernatants were collected 2 days after infection, and the viral RNA was purified using RNeasy kit (Qiagen), and stored at −80 °C until use. The concentration of the viral RNA was determined from the A_260_ value measured using a NanoDrop spectrophotometer. On the day of the opn-SATORI assay, viral RNA was thawed, diluted in Ltr buffer, and heated at 90 °C for 5 min.

### SARS-CoV-2 patient samples

Patient samples were obtained from Tokyo Medical and Dental University Hospital. Human nasopharyngeal swab samples were collected, and RNA was purified using the QIAamp Viral RNA Mini Kit (Qiagen) according to the manufacturer’s protocol. The viral RNA was eluted in nuclease-free water, and stored at −80 °C until use. On the day of the opn-SATORI assay, viral RNA was thawed, and heated at 90 °C for 5 min. RT-qPCR was performed using the extracted RNA with the QuantStudio 3 (Thermo Fisher Scientific) and Ampdirect™ 2019-nCoV Detection Kit with a primer set targeting N-gene region of SARS-CoV-2 RNA (SHIMADZU). The SARS-CoV-2 RNA purified from VeroE6/TMORSS2 cells (Wuhan strain) was used as a reference to obtain a calibration curve of RT-qPCR Ct values and the copy number of SARS-CoV-2 RNA. The obtained Ct values and copy numbers of SARS-CoV-2 RNA from patients are summarized in Supplementary Table 6. The whole viral genome sequences of SARS-CoV-2 from patients were analyzed using MiSeq (Illumina), and the information on the mutations and the lineages are summarized in Supplementary Table 6. This research was approved by Tokyo medical and dental university (approval ID number M2020-004) with informed consent from participants.

### opn-SATORI

opn-SATORI was built by incorporating a custom-made automatic dispenser machine (BioTec) into a confocal fluorescence microscope (A1HD25, Nikon), equipped with a 20×objective lens (NA = 0.75), 488 nm and 640 nm lasers, and a motorized XY scanning stage. The automatic dispenser machine can attach and remove pipette tips at programmed positions, and aspirate and discharge solutions at set rates. Thus, in this system, all operations of the SATORI assay, including mixing of Cas13a-crRNA and target RNA solutions, introducing the mixture into microchamber devices, and acquisition of fluorescent images, are fully automated.

The assay solution for SATORI (solution A) for a single assay was prepared as follows. To prepare Cas13a-crRNA complexes, a mixture of 0.7 μL of Cas13a (20 μM), 1.4 μL of crRNA and 2.6 μL of buffer G (20 mM HEPES-KOH (pH 6.8), 60 mM NaCl, 6 mM MgCl_2_ and 50 μM Triton X-100) was incubated at 37 °C for 10 min. Next, 4.7 μL of the Cas13a-crRNA solution was mixed with 18.7 μL of buffer G containing 15 μM FQ reporter (Integrated DNA Technologies) and 75 μM Alexa Fluor™ 647 C_2_ maleimide (Thermo Scientific), and stored at −80 °C until use.

Before starting the SATORI assays, frozen solution A was thawed at room temperature. Target RNA was prepared in Ltr buffer (20 mM HEPES-KOH (pH 7.5), 100 mM KCl, 10 mM MgCl_2_ and 50 μM Triton X-100), Lwa buffer (20 mM HEPES-KOH (pH 6.8), 60 mM NaCl, 6 mM MgCl_2_ and 50 μM Triton X-100) or Lbu buffer (20 mM HEPES-KOH (pH 6.8), 50 mM KCl, 5 mM MgCl_2_, 5% glycerol and 50 μM Triton X-100). Solution A, target RNA solution and mineral oil (M5904, Sigma-Aldrich) were added into plastic tubes and placed in the sample holder of the dispenser machine. For the SATORI assay, 20 μL of solution A was first mixed with 100 μL of the target RNA solution. After 1 min of incubation, 105 μL of the mixture was dropped onto a CD-based device and pipetted repeatedly for 1 min. Of the 105 μL of solution on the CD device, 95 μL was removed, and 50 μL mineral oil was added at a rate of 5 μL s^−1^ to seal the microchamber. The excess solution A and mineral oil left on the CD were removed. After 1 min of incubation, fluorescence tiling images were recorded for 25 stages at 2 min intervals. The focus was automatically adjusted based on the fluorescence intensity of Alexa Fluor™ 647 C_2_ maleimide in the microchambers.

For opn-SATORI assays coupled with magnetic beads, biotinylated LtrCas13a was prepared by incubating LtrCas13a with NHS-dPEG_4_-biotin (10200, Quanta BioDesign) in buffer E at a molar ratio of ~1:5 for 3 h at room temperature. The assay solution for opn-SATORI (solution B) was prepared using biotin-labeled Cas13a-crRNA complexes in the same manner as mentioned above.

Before the opn-SATORI assay, frozen solution B was thawed at room temperature and diluted 10-fold with buffer G containing 12 μM FQ reporter and 60 μM Alexa Fluor™ 647 C_2_ maleimide (solution C). Magnetic beads (Dynabeads MyOne Streptavidin T1, Veritas) were washed three times with buffer H (20 mM HEPES-KOH (pH 7.5), 100 mM KCl, 10 mM MgCl_2_ and 500 μM Triton X-100), and adjusted to a concentration of 0.5 mg mL^−1^ with buffer H. Solution C, target RNA solution, magnetic bead suspension and mineral oil were added to plastic tubes and placed in the tube holder of the automatic dispenser machine.

For the opn-SATORI assay, 20 μL of solution C and 100 μL of the target RNA solution were mixed, and 20 μL of the magnetic bead solution was then added. After 3 min of incubation, 105 μL of the mixture was dropped onto a CD-based device and pipetted for 10 s. A neodymium magnet (HXN10-10, Misumi) was placed directly under the device via the automated objective lens turret of the microscope. Of the 105 μL of solution on the CD device, 95 μL was removed, and subsequently 50 μL of mineral oil was added at a rate of 5 μL s^−1^. Excess solution C and mineral oil remaining on the CD were removed. The neodymium magnet was placed away from the device, and a 20× objective lens was subsequently placed under the device. After 1 min of incubation, fluorescence tiling images were recorded for 25 stages at 2 min intervals.

### Single-molecule analysis of the *trans-*cleavage activity of Cas13a

To measure the rates of *trans*-cleavage by Cas13a at the single-molecule level, mixtures of solution A and 300 fM SARS-CoV2 N-gene were introduced into the microchambers. Immediately after sealing with mineral oil, fluorescence images were recorded at 3 s intervals. The number of cleaved FQ reporters was calculated based on the calibration curve of the mean fluorescence intensity in each microchamber and the concentration of fluorescein-conjugated ssRNA (56-FAM/rUrUrUrUrU) (Supplementary Fig. 5d).

### Data analysis

The images obtained were automatically analyzed during the opn-SATORI assay based on a custom-made macro program in NIS-Elements software (Nikon) as follows: the ROIs of the microchambers for the acquired images were set by thresholding the intensity of Alexa Fluor™ 647 C_2_ maleimide in the red channel. At this stage, background noise was removed by judging the size and circularity of the ROI. The average intensity of the green channel in each microchamber was extracted. For SATORI assays, the number of positive chambers was defined as the number of chambers with a green intensity greater than 1500. For opn-SATORI assays coupled with magnetic beads, the number of positive chambers was defined as the number of chambers with a green intensity greater than 1200.

The analytical limit of detection (LoD) was defined as follows. The number of positive chambers obtained with different concentrations of the target RNA was fitted to a linear curve. The mean + 3 S.D. value for the number of positive chambers obtained without target RNA was determined, and the crossing point of the linear curve and the mean + 3 S.D. was then determined. The concentration corresponding to the crossing point is defined as the LoD value.

To discriminate the SARS-CoV-2 variants, opn-SATORI was performed using crRNA complementary to the conventional SARS-CoV-2 or the mutant strain, and the ratio of the number of positive chambers obtained with each crRNA was calculated. The presence of amino acid mutations in the samples was determined based on a ratio value greater than 1.0.

### Back-of-the-Envelope check for Michaelis–Menten analysis

To evaluate the validity of the enzyme kinetics parameters of Cas13a obtained in the single-molecule measurements, a simple back-of-the-envelope calculation was conducted as follows^19^. Briefly, if the kinetics data are correct, the nondimensional parameters *α*, *β*, and *γ* (defined below) should be less than one. *α* was calculated as the ratio of the amount of cleaved FQ-reporters at time ${t}_{{{{{{\rm{lin}}}}}}}$ to the initial concentration of the uncleaved reporter ${S}_{0}$.

1 $${{\alpha }}=\frac{v{t}_{{{{{{\rm{lin}}}}}}}}{{{{{{{\rm{S}}}}}}}_{0}} \; < \; 1$$

here, *v* is the *trans*-cleavage activity of Cas13a obtained in our measurements (Supplementary Fig. 5g), and *t*_lin_ is the duration at which fluorescence intensity was increased linearly (40 s in Supplementary Fig. 5a–c). β was calculated by normalizing the obtained *trans*-cleavage activity ($v$) by ${v}_{{{\max }}}$;

2 $${{\beta }}=\frac{v}{{v}_{{{\max }}}}=\frac{v}{{k}_{{{{{{\rm{cat}}}}}}}{E}_{0}} \; < \; 1$$

$${E}_{0}=\frac{1}{V{N}_{A}}=49({{{{{\rm{pM}}}}}})$$

The positive chamber contained only one molecule of Cas13a-crRNA-tgRNA, and$\,{E}_{0}$ was calculated as a function of the chamber volume (*V*: 34 fL) and Avogadro’s constant (*N*_A_: 6 × 10^23^). The turnover rate (*k*_cat_) and Michaelis–Menten constant (*K*_m_) were determined by fitting to the Michaelis–Menten equation (Supplementary Fig. 5g). *γ* was calculated as the ratio of ${t}_{{{{{{\rm{lin}}}}}}}$ to the reaction time scale ($\tau$ $=$ ${K}_{{{{{{\rm{m}}}}}}}{k}_{{cat}}^{-1}{E}_{0}^{-1}$);

3 $${{\gamma }}=\frac{{t}_{{{{{{\rm{lin}}}}}}}}{\tau }=\frac{{t}_{{{{{{\rm{lin}}}}}}}{k}_{{{{{{\rm{cat}}}}}}}{{{{{{\rm{E}}}}}}}_{0}}{{K}_{{{{{{\rm{m}}}}}}}} \; < \; 1$$

### Statistics and reproducibility

All the measurements, described in this paper, were taken from distinct samples, and all experiments performed on the paper were successfully replicated more than three times.

### Reporting summary

Further information on research design is available in the Nature Research Reporting Summary linked to this article.

## Supplementary information

Supplemental Materials

Description of Additional Supplementary Files

Supplementary Data 1

Reporting Summary

## Data Availability

All source data used for generating graphs and charts in main and supplementary figures are included in Supplementary Data 1. Any other data are available from the corresponding authors on reasonable request.
