# Supplementary material for: Automated amplification-free digital RNA detection platform for rapid and sensitive SARS-CoV-2 diagnosis
Source: Commun Biol. 2022 May 26;5:473. doi: 10.1038/s42003-022-03433-6 (PMC9132978; doi:10.1038/s42003-022-03433-6)
Supplement: Supplementary file 1 — Supplemental Materials [file 42003_2022_3433_MOESM1_ESM.pdf]

Supplementary Materials for

Automated amplification-free digital RNA detection platform for rapid and sensitive SARS-CoV-2 diagnosis

Hajime Shinoda, Tatsuya Iida, Asami Makino, Mami Yoshimura, Junichiro Ishikawa, Jun Ando, Kazue Murai, Katsumi Sugiyama, Masahiro Nakano, Yukiko Muramoto, Kotaro Kiga, Longzhu Cui, Osamu Nureki, Hiroaki Takeuchi, Takeshi Noda, Hiroshi Nishimasu*, Rikiya Watanabe*

*Corresponding authors. nisimasu@g.ecc.u-okyo.ac.jp (H.N.), rikiya.watanabe@riken.jp (R.W.)

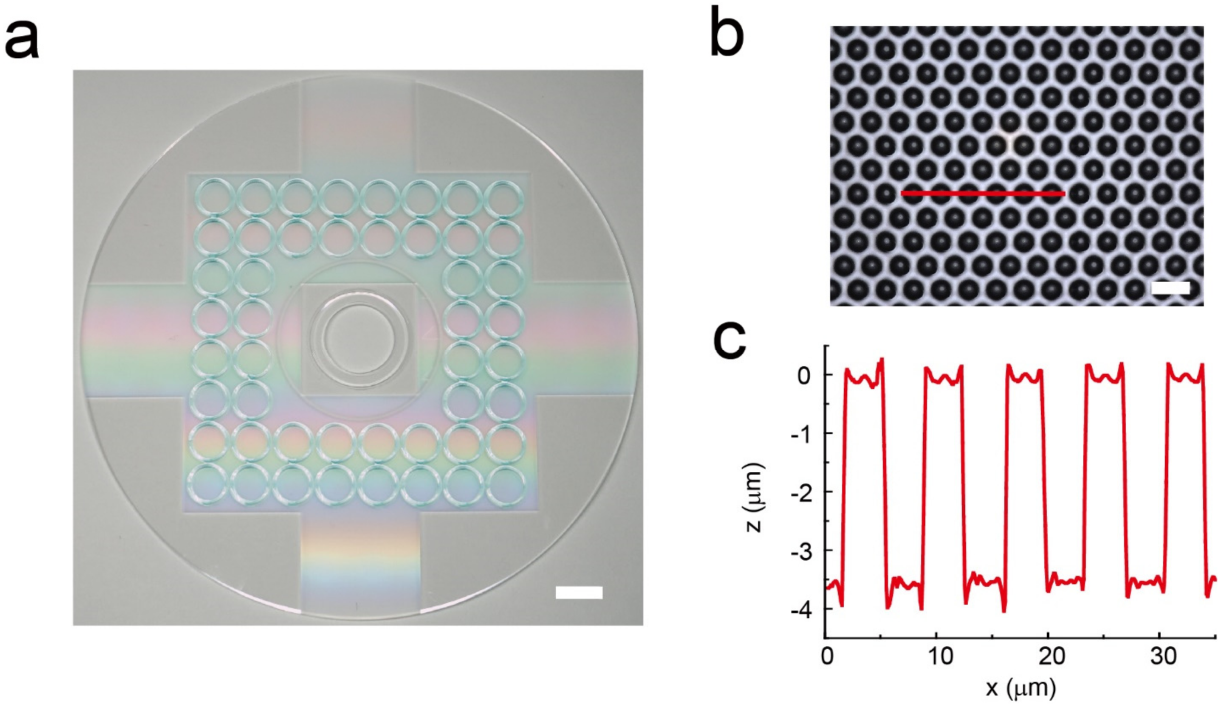

Supplementary Figure 1. CD-based microchamber device
(a) Picture of a CD-based microchamber device. Microchambers are located in the area where the interference pattern appears on the CD. The ring-shaped enclosures on the CD were fabricated using UV-curing acrylic resin. Scale bar, 1 cm. (b) Surface image of the microchambers, acquired using 3D confocal laser microscope. Scale bar, 10 µm. (c) Line profile of the cross-section on the red line in (b). The average diameter, height and volume are ~3.5 µm, ~3.5 µm and ~30 fL, respectively.

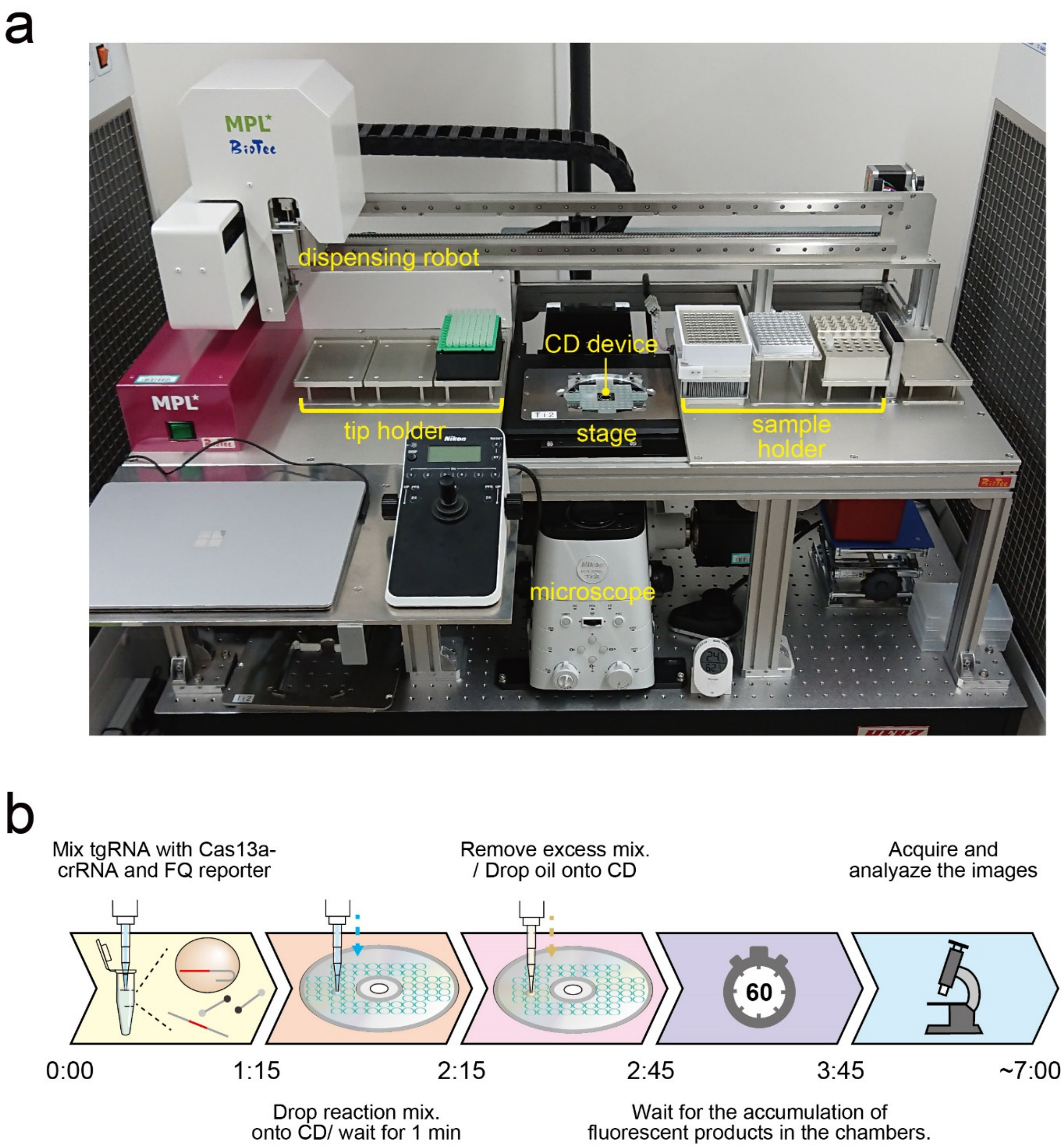

Supplementary Figure 2. Automated microscope equipped with a dispensing robot
(a) Picture of an automated digital RNA detection platform. (b) Time scheme of the assay from sample mixing to image analysis.

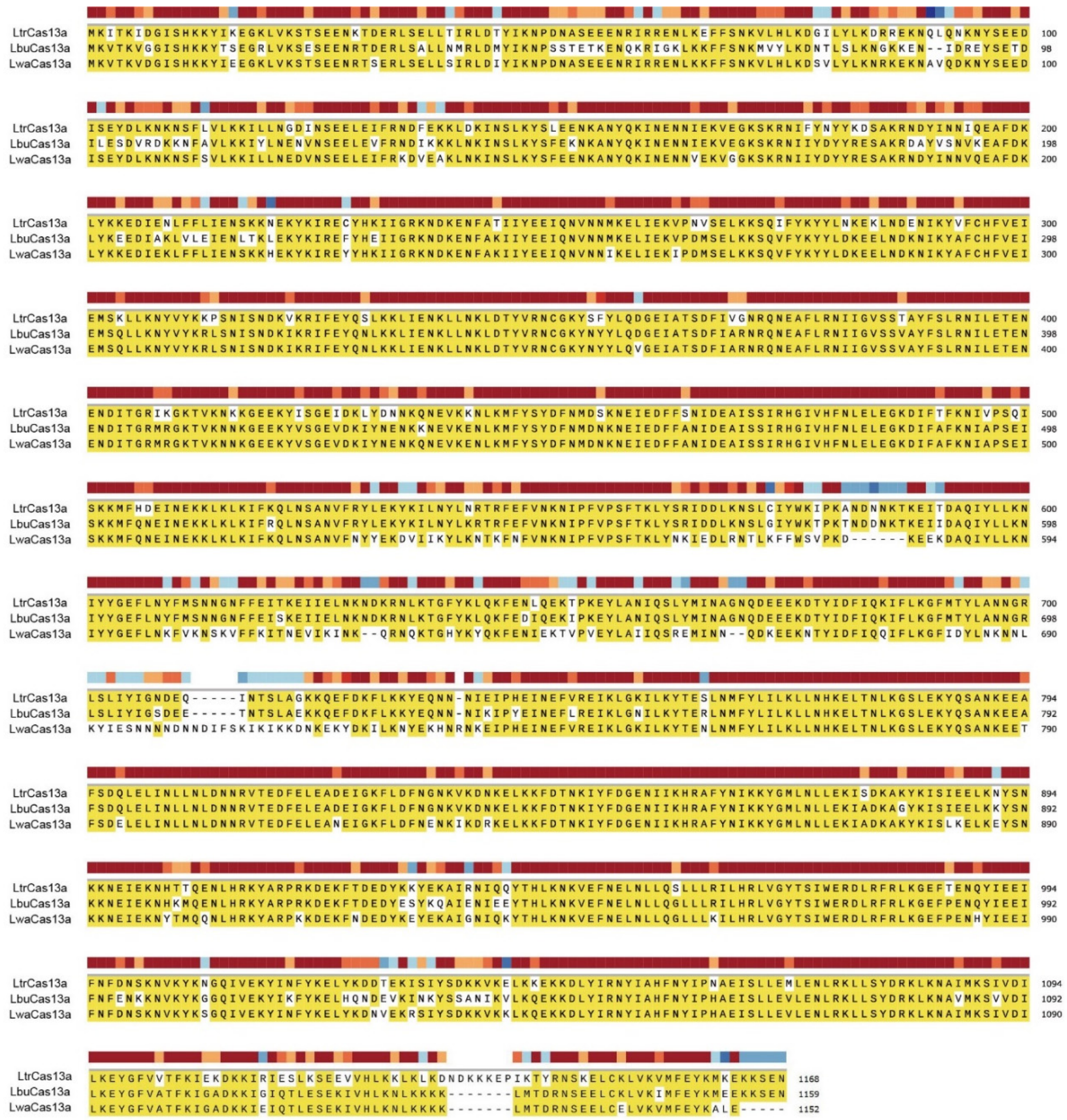

Supplementary Figure 3. Sequence alignment of LtrCas13a, LbuCas13a, and LwaCas13a
The amino acid sequences were aligned using a ClustalW software. LtrCas13a shares 88% and 83% sequence identity with LwaCas13a and LbuCas13a, respectively.

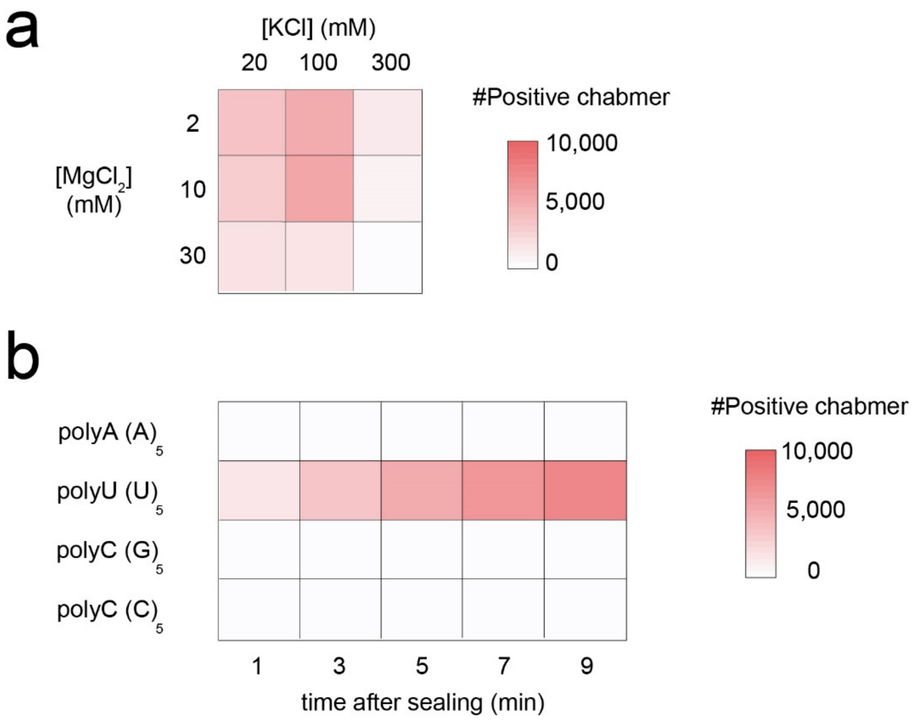

Supplementary Figure 4. Biochemical characterization of LtrCas13a
(a) Buffer optimization for LtrCas13a. The optimal concentration of KCl and $MgCl_2$ was screened using 20 mM HEPES-KOH (pH 7.5) buffer containing 50 μM Triton X-100 for LtrCas13a with crRNA-Ltr-N2. In the presence of 100 mM KCl and 10 mM $MgCl_2$ (Ltr buffer), the number of positive chambers increased most rapidly. Data indicate mean (n = 3 technical replicates). (b) Heatmap showing nucleotide preferences in the *trans*-cleavage activity of LtrCas13a with a crRNA-Ltr-N2 in Ltr buffer (n = 3 technical replicates).

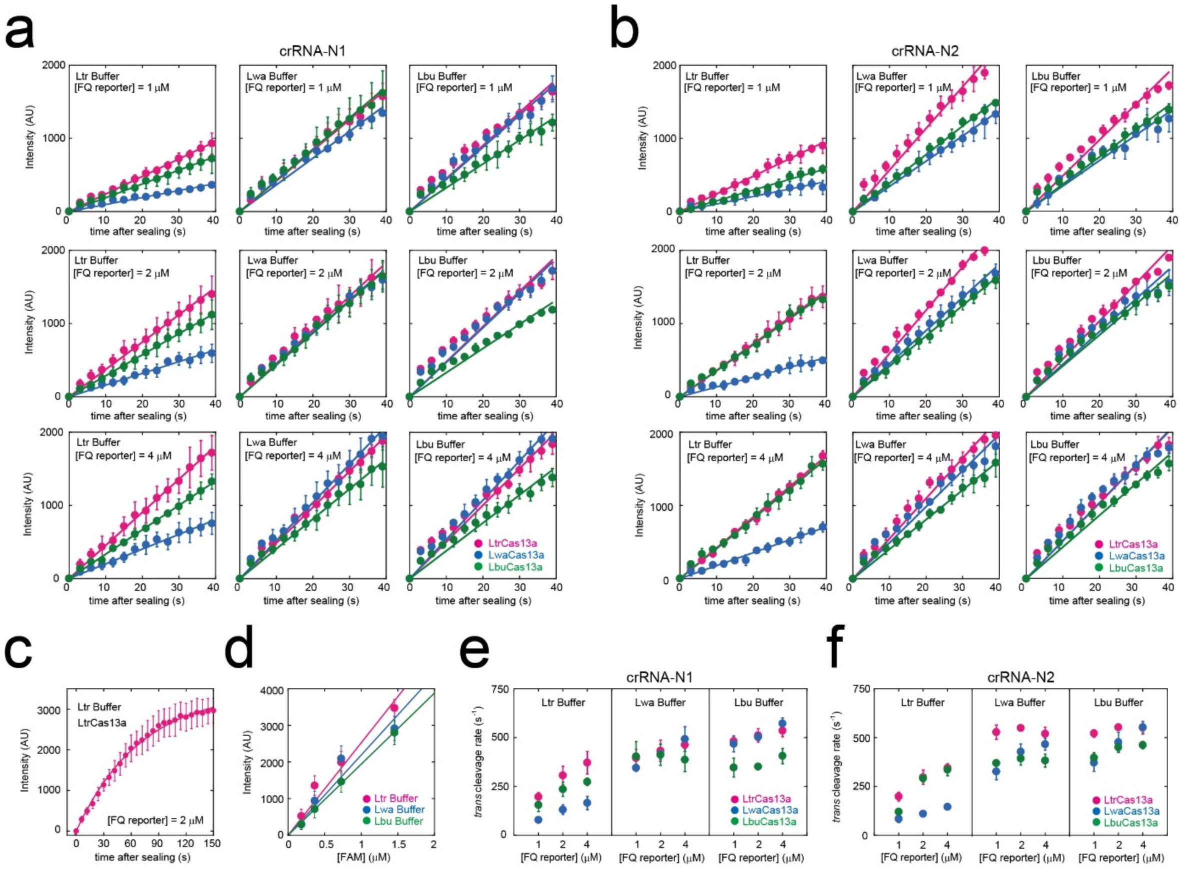

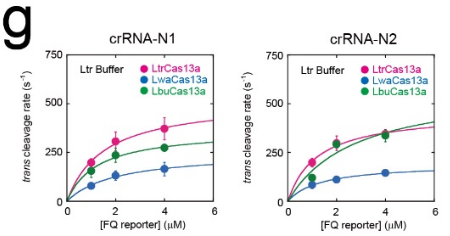

h

Protein	LtrCas13a		LwaCas13a		LbuCas13a	
crRNA	N1	N2	N1	N2	N1	N2
v (s^{-1})	306	297	131	110	236	292
k_{cat} (s^{-1})	521	457	253	192	366	596
K_m (μM)	1.54	1.21	2.04	1.35	1.26	2.80
α	0.30	0.29	0.13	0.11	0.23	0.29
β	0.59	0.65	0.52	0.58	0.64	0.49
γ	0.39	0.48	0.13	0.16	0.37	0.21

Supplementary Figure 5. Comparison of *trans*-cleavage activities of the Cas13a enzymes at single-molecule levels

(a, b) Time courses showing fluorescence increase of single Cas13a-crRNA-tgRNA molecules at different concentrations of FQ reporters. Fluorescence images were recorded immediately after sealing the microchambers with mineral oil. The solid lines indicate linear regressions. (c) Time-courses of fluorescence increases upon *trans*-cleavage of FQ reporters by LtrCas13a-crRNA-tgRNA in Ltr buffer with 2 μM FQ reporters. (d) Calibration curve of the maximum intensity in each chamber to FAM-conjugated ssRNA concentration. The solid lines indicate linear regressions. (e-g) Rate of *trans*-cleavage of LtrCas13a-crRNA-tgRNA, LwaCas13a-crRNA-tgRNA and LbuCas13a-crRNA-tgRNA at the single-molecule level. Data were obtained with crRNA-N1 (e) or crRNA-N2 (f). Data obtained in Ltr buffer were fitted to a Michaelis-Menten equation (g). Data show the mean ± S.D. (h) Parameter for *trans*-cleavage of Cas13a orthologs in Ltr buffer. k_{cat} and K_m were determined from graphs (g). The accuracy of the data was verified by a simple back-of-the-envelope calculation based on a Michaelis-Menten equation using *trans*-cleavage activity (v) with 2 μM FQ reporter, where the parameters α, β, and γ defined in this calculation become less than 1 for correct data ("Methods"). (n=3 technical replicates for a-b, d-g).

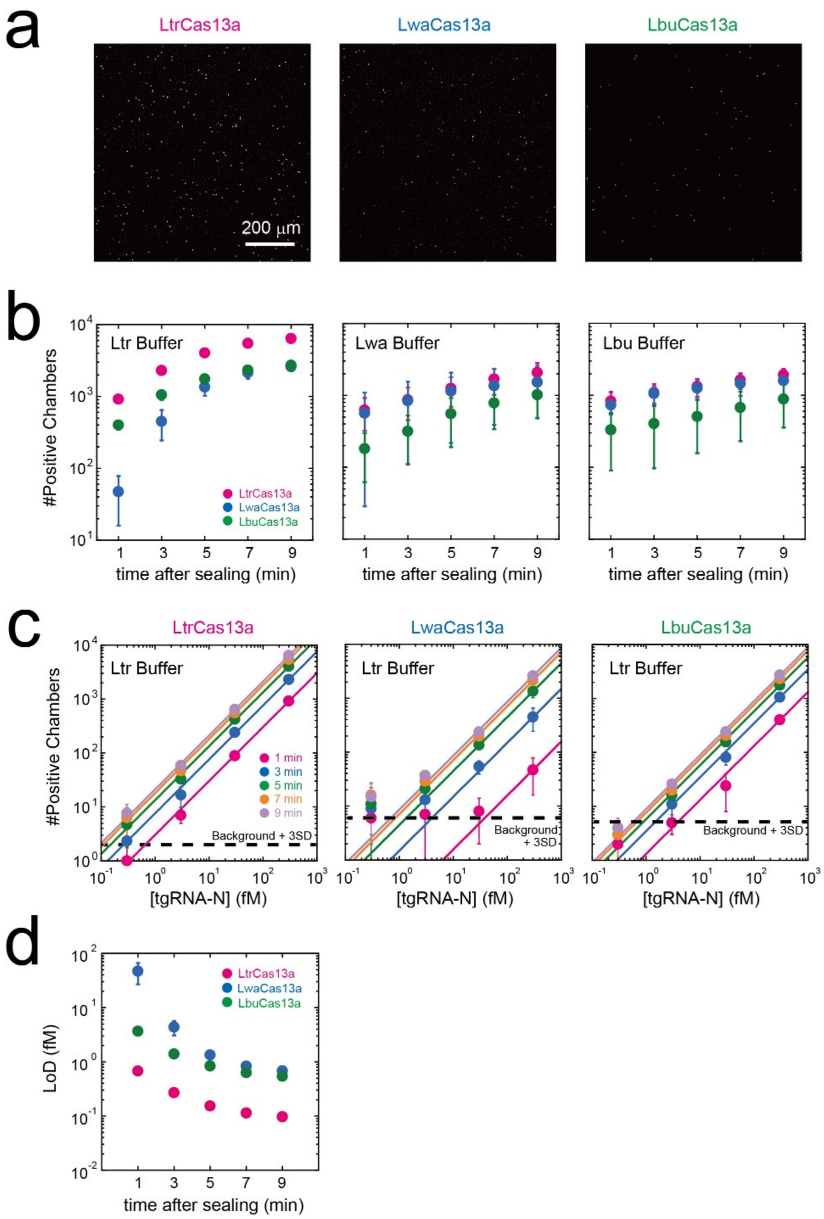

Supplementary Figure 6. Screening for the optimal Cas13a enzyme and buffer conditions for the opn-SATORI assay

(a) Representative fluorescence images obtained with LtrCas13a, LwaCas13a, or LbuCas13a in the Ltr buffer, and using the crRNA-N1 and the N-gene RNA (300 fM). (b) Time courses for the number of positive chambers obtained with LtrCas13a, LwaCas13a, or LbuCas13a with their respective optimized buffers, using the crRNA-N1 and the N-gene RNA (300 fM). (c) The number of the positive chambers obtained with the three Cas13a enzymes in the Ltr buffer at different concentrations of the N-gene RNA. The number of positive chambers obtained at the indicated time after sealing the device is shown. The solid lines indicate linear regressions. (d) LoD values for opn-SATORI with the Cas13a enzymes in the Ltr buffer. Data show the mean ± S.D. (n = 3 technical replicates).

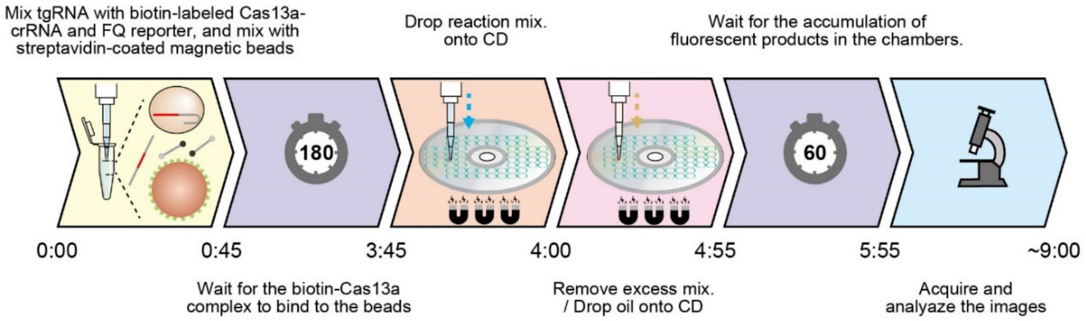

Supplementary Figure 7. Time scheme of opn-SATORI operation with magnetic beads from sample mixing to image analysis

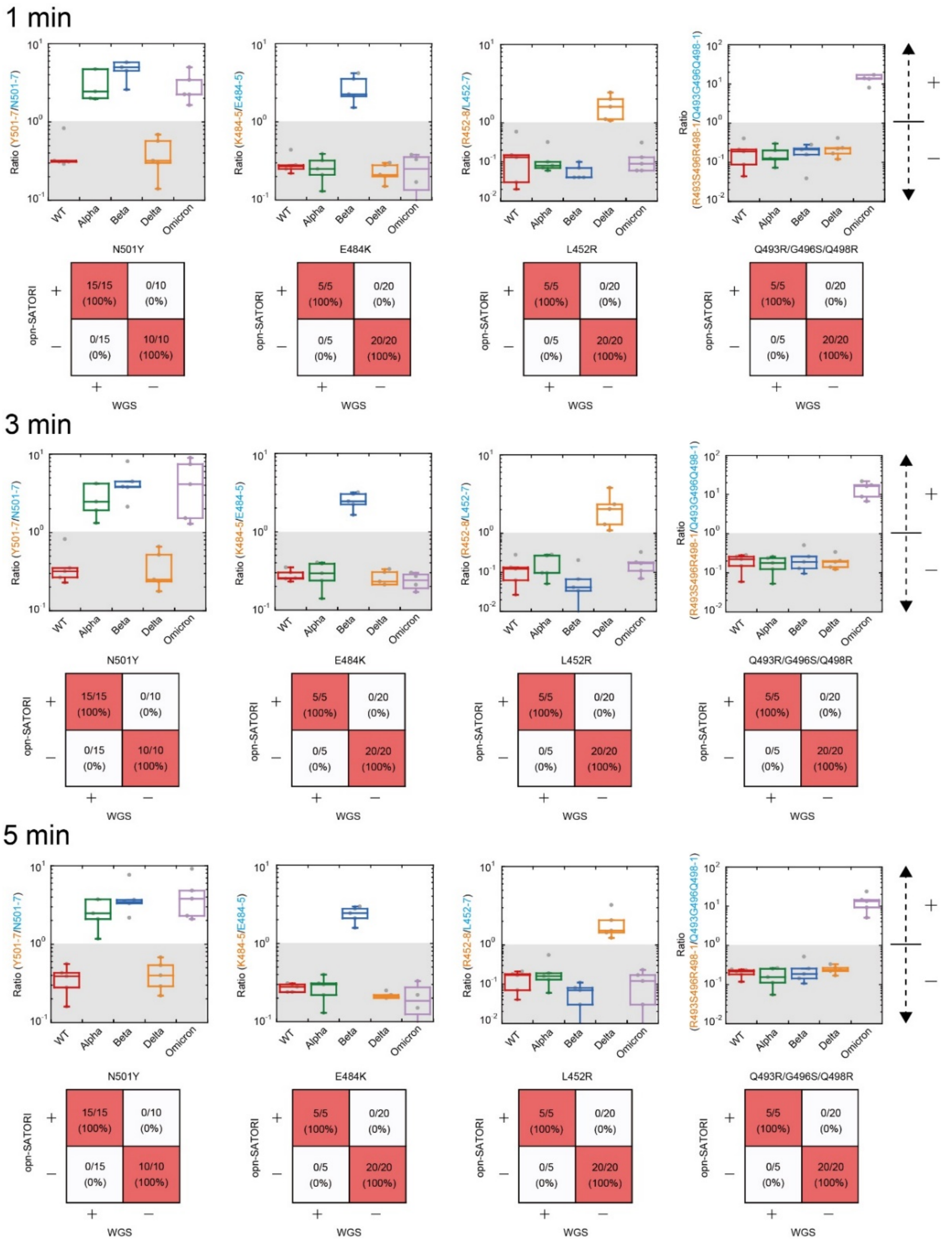

Supplementary Figure 8. Discrimination of mutations in the S-gene of the SARS-CoV-2 variants
The ratio values were obtained at 1 min (top), 3 min (middle), and 5 min (bottom) after the device was sealed with oil (n = 5 technical replicates). Wuhan-Hu1, B.1.1.7, B.1.351, B.1.617, and B.1.1.529 were used as the WT, α, β, δ, and o variants, respectively. The definition of having mutations is that the ratio value is greater than 1.0.

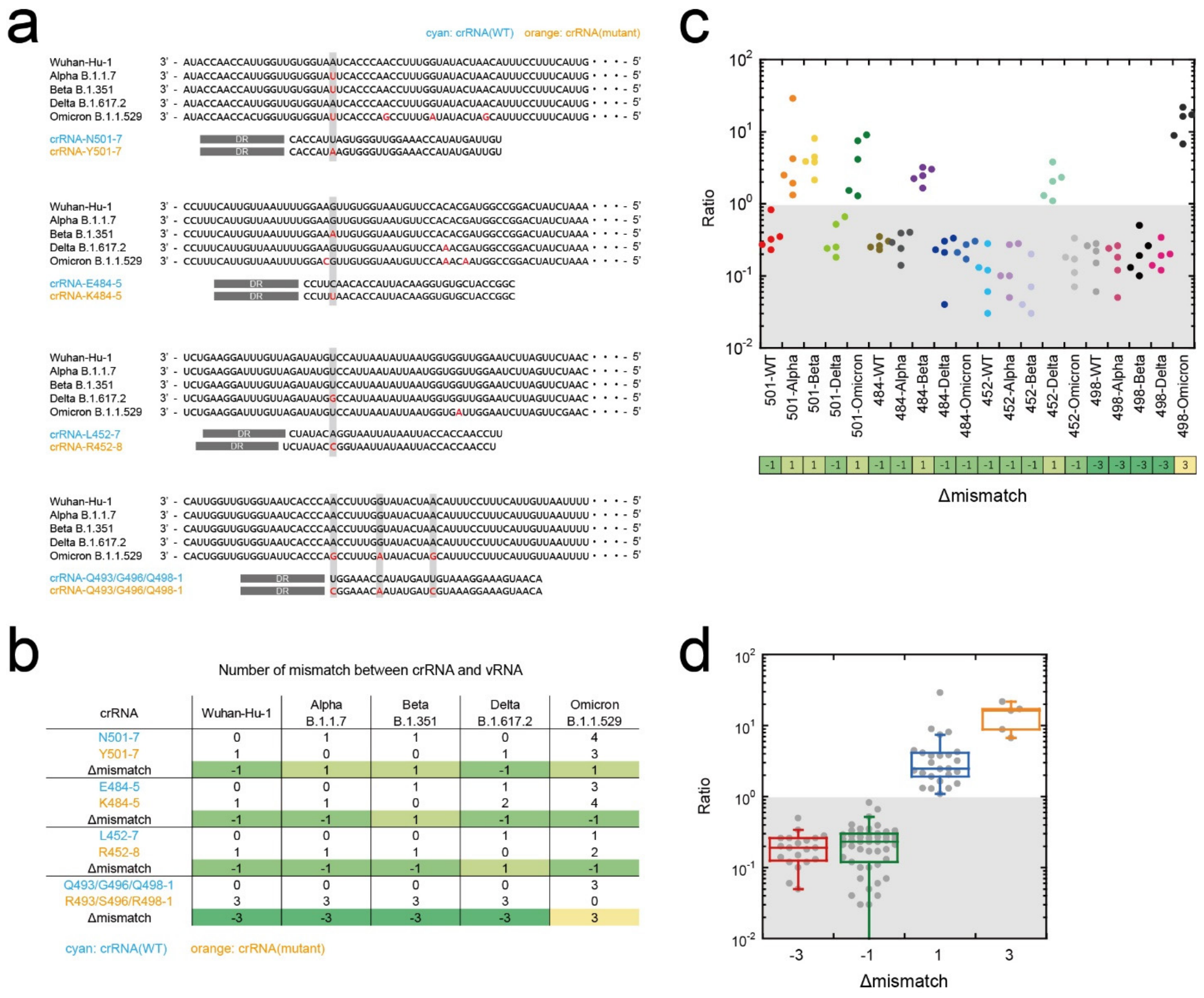

crRNA	Wuhan-Hu-1	Alpha B.1.1.7	Beta B.1.351	Delta B.1.617.2	Omicron B.1.1.529
N501-7	0	1	1	0	4
Y501-7	1	0	0	1	3
Δmismatch	-1	1	1	-1	1
E484-5	0	0	1	1	3
K484-5	1	1	0	2	4
Δmismatch	-1	-1	1	-1	-1
L452-7	0	0	0	1	1
R452-8	1	1	1	0	2
Δmismatch	-1	-1	-1	1	-1
Q493/G496/Q498-1	0	0	0	0	3
R493/S496/R498-1	3	3	3	3	0
Δmismatch	-3	-3	-3	-3	3

Supplementary Figure 9. Effect of the number of mismatches on the discrimination of the variants
(a) Sequence alignment of crRNA and wild-type or mutant SARS-CoV-2 RNAs. (b) The number of mismatches between crRNA and SARS-CoV-2 RNAs. Δ mismatch represents differences in the number of mismatches between the WT and mutant crRNAs against the S-genes. (c, d) The ratio of the number of positive chambers obtained with crRNA pairs (N501-7/Y501-7, E484-5/K484-5, L452-7/R452-8 or Q493G496Q498-1/R493S496R498-1). The definition of having mutations is that the ratio value is greater than 1.0.

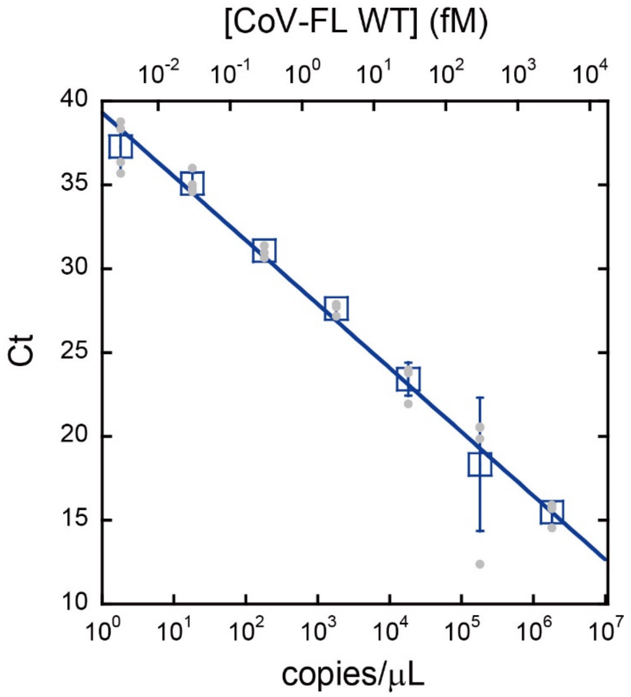

Supplementary Figure 10. Calibration curve of Ct values and copy number for SARS-CoV-2 whole-genome RNA (SARS-CoV-2/Wuhan-Hu-1)

Copy numbers of the viral RNAs were determined from the A_{260} value measured using a NanoDrop spectrophotometer. Data were fitted to a logarithmic equation (n = 4 technical replicates).

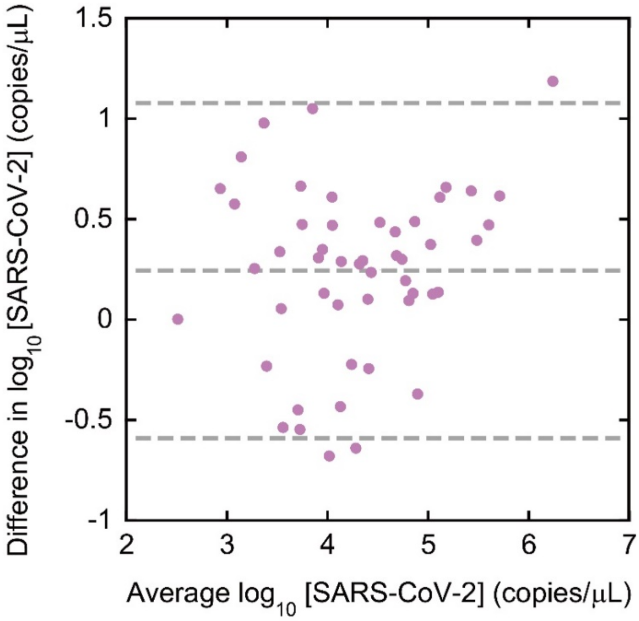

Supplementary Figure 11. Bland-Altman analysis between SARS-CoV-2 RNA concentration determined by opnSATORI and RT-qPCR.
The center dashed line represents the mean difference, and the upper and the lower dashed lines represent 95% limits of agreement.

	WT	Alpha	JP	Beta	Delta	Omicron
N501Y	−	+	−	+	−	+
E484K	−	−	+	+	−	−
L452R	−	−	−	−	+	−
Q493R/G496S/Q498R	−	−	−	−	−	+

Supplementary Figure 12. Correspondence table between SARS-CoV-2 variants and mutation status

Supplementary Table 1. Chemical-synthesis-crRNA sequences used in this study.

Name	Sequence
Ltr-N1	GGAUUUAGAGUACCCCAAAAAUGAAGGGGACUAAAAC AAGGUCUUCCUUGCCAUGUUGAGUGAGAGCGG
Ltr-N2	GGAUUUAGAGUACCCCAAAAAUGAAGGGGACUAAAAC UUGGCAAUGUUGUUCCUUGAGGAAGUUGUAGC
Lwa-N1	GGAUUUAGACUACCCCAAAAACGAAGGGGACUAAAAC AAGGUCUUCCUUGCCAUGUUGAGUGAGA
Lwa-N2	GGAUUUAGACUACCCCAAAAACGAAGGGGACUAAAAC UUGGCAAUGUUGUUCCUUGAGGAAGUUG
Lbu-N1	GGACCACCCCAAAAAUGAAGGGGACUAAAACA AAGGUCUUCCUUGCCAUGUUGAGUGAGA
Lbu-N2	GGACCACCCCAAAAAUGAAGGGGACUAAAACA UUGGCAAUGUUGUUCCUUGAGGAAGUUG
Ltr-S-E484-5	GGAUUUAGAGUACCCCAAAAAUGAAGGGGACUAAAAC CCUUCAACACCAUUACAAGGUGUGCUACCGGC
Ltr-S-K484-5	GGAUUUAGAGUACCCCAAAAAUGAAGGGGACUAAAAC CCUUUAACACCAUUACAAGGUGUGCUACCGGC
Ltr-S-N501-7	GGAUUUAGAGUACCCCAAAAAUGAAGGGGACUAAAAC CACCAUUAGUGGGUUGGAAACCAUAUGAUUGU
Ltr-S-Y501-7	GGAUUUAGAGUACCCCAAAAAUGAAGGGGACUAAAAC CACCAUAAGUGGGUUGGAAACCAUAUGAUUGU
Ltr-S-L452-7	GGAUUUAGAGUACCCCAAAAAUGAAGGGGACUAAAAC CUAUACAGGUAAUUAUAAUUACCACCAACCUU
Ltr-S-R452-8	GGAUUUAGAGUACCCCAAAAAUGAAGGGGACUAAAAC UCUAUACCGGUAAUUAUAAUUACCACCAACCU
Ltr-S-Q493/G496/Q498-1	GGAUUUAGAGUACCCCAAAAAUGAAGGGGACUAAAAC UGGAAACCAUAUGAUUGUAAAGGAAAGUAACA
Ltr-S-R493/S496/R498-1	GGAUUUAGAGUACCCCAAAAAUGAAGGGGACUAAAAC CGGAAACUAUAUGAUCGUAAAGGAAAGUAACA

Supplementary Table 2. FQ reporters used in this study.

Name	Sequence
FQ reporter (FAM-U_5-Q)	5′-/56-FAM/rUrUrUrUrU/3IABkFQ/-3′
FAM-A_5-Q	5′-/56-FAM/rArArArArA/3IABkFQ/-3′
FAM-C_5-Q	5′-/56-FAM/rCrCrCrCrC/3IABkFQ/-3′
FAM-G_5-Q	5′-/6FAM/rGrGrGrGrG/BHQ/-3′
FAM without quencher	5′-/56-FAM/rUrUrUrUrU/-3′

Supplementary Table 3. IVT-crRNA sequences used in this study.

Name	Sequence
Ltr-S-E484-1	GGAUUUAGAGUACCCCAAAAAUGAAGGGGACUAAAAC CAACACCAUUACAAGGUGUGCUACCGGCCUGA
Ltr-S-E484-2	GGAUUUAGAGUACCCCAAAAAUGAAGGGGACUAAAAC UCAACACCAUUACAAGGUGUGCUACCGGCCUG
Ltr-S-E484-3	GGAUUUAGAGUACCCCAAAAAUGAAGGGGACUAAAAC UUCAACACCAUUACAAGGUGUGCUACCGGCCU

Ltr-S-E484-4	GGAUUUAGAGUACCCCAAAAAUGAAGGGGACUAAAAC<br>CUUCAACACCAUUACAAGGUGUGCUACCGGCC
Ltr-S-E484-5	GGAUUUAGAGUACCCCAAAAAUGAAGGGGACUAAAAC<br>CCUUCAACACCAUUACAAGGUGUGCUACCGGC
Ltr-S-E484-6	GGAUUUAGAGUACCCCAAAAAUGAAGGGGACUAAAAC<br>ACCUUCAACACCAUUACAAGGUGUGCUACCGG
Ltr-S-E484-7	GGAUUUAGAGUACCCCAAAAAUGAAGGGGACUAAAAC<br>AACCUUCAACACCAUUACAAGGUGUGCUACCG
Ltr-S-E484-8	GGAUUUAGAGUACCCCAAAAAUGAAGGGGACUAAAAC<br>AAACCUUCAACACCAUUACAAGGUGUGCUACC
Ltr-S-E484-9	GGAUUUAGAGUACCCCAAAAAUGAAGGGGACUAAAAC<br>AAAACCUUCAACACCAUUACAAGGUGUGCUAC
Ltr-S-E484-10	GGAUUUAGAGUACCCCAAAAAUGAAGGGGACUAAAAC<br>UAAAACCUUCAACACCAUUACAAGGUGUGCUA
Ltr-S-K484-1	GGAUUUAGAGUACCCCAAAAAUGAAGGGGACUAAAAC<br>UAACACCAUUACAAGGUGUGCUACCGGCCUGA
Ltr-S-K484-2	GGAUUUAGAGUACCCCAAAAAUGAAGGGGACUAAAAC<br>UUAACACCAUUACAAGGUGUGCUACCGGCCUG
Ltr-S-K484-3	GGAUUUAGAGUACCCCAAAAAUGAAGGGGACUAAAAC<br>UUUAACACCAUUACAAGGUGUGCUACCGGCCU
Ltr-S-K484-4	GGAUUUAGAGUACCCCAAAAAUGAAGGGGACUAAAAC<br>CUUUAACACCAUUACAAGGUGUGCUACCGGCC
Ltr-S-K484-5	GGAUUUAGAGUACCCCAAAAAUGAAGGGGACUAAAAC<br>CCUUUAACACCAUUACAAGGUGUGCUACCGGC
Ltr-S-K484-6	GGAUUUAGAGUACCCCAAAAAUGAAGGGGACUAAAAC<br>ACCUUUAACACCAUUACAAGGUGUGCUACCGG
Ltr-S-K484-7	GGAUUUAGAGUACCCCAAAAAUGAAGGGGACUAAAAC<br>AACCUUUAACACCAUUACAAGGUGUGCUACCG
Ltr-S-K484-8	GGAUUUAGAGUACCCCAAAAAUGAAGGGGACUAAAAC<br>AAACCUUUAACACCAUUACAAGGUGUGCUACC
Ltr-S-K484-9	GGAUUUAGAGUACCCCAAAAAUGAAGGGGACUAAAAC<br>AAAACCUUUAACACCAUUACAAGGUGUGCUAC
Ltr-S-K484-10	GGAUUUAGAGUACCCCAAAAAUGAAGGGGACUAAAAC<br>UAAAACCUUUAACACCAUUACAAGGUGUGCUA
Ltr-S-N501-1	GGAUUUAGAGUACCCCAAAAAUGAAGGGGACUAAAAC<br>UAGUGGGUUGGAAACCAUAUGAUUGUAAAGGA
Ltr-S-N501-2	GGAUUUAGAGUACCCCAAAAAUGAAGGGGACUAAAAC<br>UUAGUGGGUUGGAAACCAUAUGAUUGUAAAGG
Ltr-S-N501-3	GGAUUUAGAGUACCCCAAAAAUGAAGGGGACUAAAAC<br>AUUAGUGGGUUGGAAACCAUAUGAUUGUAAAG
Ltr-S-N501-4	GGAUUUAGAGUACCCCAAAAAUGAAGGGGACUAAAAC<br>CAUUAGUGGGUUGGAAACCAUAUGAUUGUAAA
Ltr-S-N501-5	GGAUUUAGAGUACCCCAAAAAUGAAGGGGACUAAAAC<br>CCAUUAGUGGGUUGGAAACCAUAUGAUUGUAA
Ltr-S-N501-6	GGAUUUAGAGUACCCCAAAAAUGAAGGGGACUAAAAC<br>ACCAUUAGUGGGUUGGAAACCAUAUGAUUGUA
Ltr-S-N501-7	GGAUUUAGAGUACCCCAAAAAUGAAGGGGACUAAAAC<br>CACCAUUAGUGGGUUGGAAACCAUAUGAUUGU
Ltr-S-N501-8	GGAUUUAGAGUACCCCAAAAAUGAAGGGGACUAAAAC<br>ACACCAUUAGUGGGUUGGAAACCAUAUGAUUG
Ltr-S-N501-9	GGAUUUAGAGUACCCCAAAAAUGAAGGGGACUAAAAC<br>AACACCAUUAGUGGGUUGGAAACCAUAUGAUU
Ltr-S-N501-10	GGAUUUAGAGUACCCCAAAAAUGAAGGGGACUAAAAC<br>CAACACCAUUAGUGGGUUGGAAACCAUAUGAU

Ltr-S-Y501-1	GGAUUUAGAGUACCCCAAAAAUGAAGGGGACUAAAAC<br>AAGUGGGUUGGAAACCAUAUGAUUGUAAAGGA
Ltr-S-Y501-2	GGAUUUAGAGUACCCCAAAAAUGAAGGGGACUAAAAC<br>UAAGUGGGUUGGAAACCAUAUGAUUGUAAAGG
Ltr-S-Y501-3	GGAUUUAGAGUACCCCAAAAAUGAAGGGGACUAAAAC<br>AUAAGUGGGUUGGAAACCAUAUGAUUGUAAAG
Ltr-S-Y501-4	GGAUUUAGAGUACCCCAAAAAUGAAGGGGACUAAAAC<br>CAUAAGUGGGUUGGAAACCAUAUGAUUGUAAA
Ltr-S-Y501-5	GGAUUUAGAGUACCCCAAAAAUGAAGGGGACUAAAAC<br>CCAUAAGUGGGUUGGAAACCAUAUGAUUGUAA
Ltr-S-Y501-6	GGAUUUAGAGUACCCCAAAAAUGAAGGGGACUAAAAC<br>ACCAUAAGUGGGUUGGAAACCAUAUGAUUGUA
Ltr-S-Y501-7	GGAUUUAGAGUACCCCAAAAAUGAAGGGGACUAAAAC<br>CACCAUAAGUGGGUUGGAAACCAUAUGAUUGU
Ltr-S-Y501-8	GGAUUUAGAGUACCCCAAAAAUGAAGGGGACUAAAAC<br>ACACCAUAAGUGGGUUGGAAACCAUAUGAUUG
Ltr-S-Y501-9	GGAUUUAGAGUACCCCAAAAAUGAAGGGGACUAAAAC<br>AACACCAUAAGUGGGUUGGAAACCAUAUGAUU
Ltr-S-Y501-10	GGAUUUAGAGUACCCCAAAAAUGAAGGGGACUAAAAC<br>CAACACCAUAAGUGGGUUGGAAACCAUAUGAU
Ltr-S-L452-1	GGAUUUAGAGUACCCCAAAAAUGAAGGGGACUAAAAC<br>AGGUAAUUAUAAUUACCACCAACCUUAGAAUC
Ltr-S-L452-2	GGAUUUAGAGUACCCCAAAAAUGAAGGGGACUAAAAC<br>CAGGUAAUUAUAAUUACCACCAACCUUAGAAU
Ltr-S-L452-3	GGAUUUAGAGUACCCCAAAAAUGAAGGGGACUAAAAC<br>ACAGGUAAUUAUAAUUACCACCAACCUUAGAA
Ltr-S-L452-4	GGAUUUAGAGUACCCCAAAAAUGAAGGGGACUAAAAC<br>UACAGGUAAUUAUAAUUACCACCAACCUUAGA
Ltr-S-L452-5	GGAUUUAGAGUACCCCAAAAAUGAAGGGGACUAAAAC<br>AUACAGGUAAUUAUAAUUACCACCAACCUUAG
Ltr-S-L452-6	GGAUUUAGAGUACCCCAAAAAUGAAGGGGACUAAAAC<br>UAUACAGGUAAUUAUAAUUACCACCAACCUUA
Ltr-S-L452-7	GGAUUUAGAGUACCCCAAAAAUGAAGGGGACUAAAAC<br>CUAUACAGGUAAUUAUAAUUACCACCAACCUU
Ltr-S-L452-8	GGAUUUAGAGUACCCCAAAAAUGAAGGGGACUAAAAC<br>UCUAUACAGGUAAUUAUAAUUACCACCAACCU
Ltr-S-L452-9	GGAUUUAGAGUACCCCAAAAAUGAAGGGGACUAAAAC<br>AUCUAUACAGGUAAUUAUAAUUACCACCAACC
Ltr-S-L452-10	GGAUUUAGAGUACCCCAAAAAUGAAGGGGACUAAAAC<br>AAUCUAUACAGGUAAUUAUAAUUACCACCAAC
Ltr-S-R452-1	GGAUUUAGAGUACCCCAAAAAUGAAGGGGACUAAAAC<br>CGGUAAUUAUAAUUACCACCAACCUUAGAAUC
Ltr-S-R452-2	GGAUUUAGAGUACCCCAAAAAUGAAGGGGACUAAAAC<br>CCGGUAAUUAUAAUUACCACCAACCUUAGAAU
Ltr-S-R452-3	GGAUUUAGAGUACCCCAAAAAUGAAGGGGACUAAAAC<br>ACCGGUAAUUAUAAUUACCACCAACCUUAGAA
Ltr-S-R452-4	GGAUUUAGAGUACCCCAAAAAUGAAGGGGACUAAAAC<br>UACCGGUAAUUAUAAUUACCACCAACCUUAGA
Ltr-S-R452-5	GGAUUUAGAGUACCCCAAAAAUGAAGGGGACUAAAAC<br>AUACCGGUAAUUAUAAUUACCACCAACCUUAG
Ltr-S-R452-6	GGAUUUAGAGUACCCCAAAAAUGAAGGGGACUAAAAC<br>UAUACCGGUAAUUAUAAUUACCACCAACCUUA
Ltr-S-R452-7	GGAUUUAGAGUACCCCAAAAAUGAAGGGGACUAAAAC<br>CUAUACCGGUAAUUAUAAUUACCACCAACCUU

Ltr-S-R452-8	GGAUUUAGAGUACCCCAAAAAUGAAGGGGACUAAAAC<br>UCUAUACCGGUAAUUAUAAUUACCACCAACCU
Ltr-S-R452-9	GGAUUUAGAGUACCCCAAAAAUGAAGGGGACUAAAAC<br>AUCUAUACCGGUAAUUAUAAUUACCACCAACC
Ltr-S-R452-10	GGAUUUAGAGUACCCCAAAAAUGAAGGGGACUAAAAC<br>AAUCUAUACCGGUAAUUAUAAUUACCACCAAC
Ltr-S-Q493/G496/Q498-1	GGAUUUAGAGUACCCCAAAAAUGAAGGGGACUAAAAC<br>UGGAAACCAUAUGAUUGUAAAGGAAAGUAACA
Ltr-S-Q493/G496/Q498-2	GGAUUUAGAGUACCCCAAAAAUGAAGGGGACUAAAAC<br>UUGGAAACCAUAUGAUUGUAAAGGAAAGUAAC
Ltr-S-Q493/G496/Q498-3	GGAUUUAGAGUACCCCAAAAAUGAAGGGGACUAAAAC<br>GUUGGAAACCAUAUGAUUGUAAAGGAAAGUAA
Ltr-S-Q493/G496/Q498-4	GGAUUUAGAGUACCCCAAAAAUGAAGGGGACUAAAAC<br>GGUUGGAAACCAUAUGAUUGUAAAGGAAAGUA
Ltr-S-Q493/G496/Q498-5	GGAUUUAGAGUACCCCAAAAAUGAAGGGGACUAAAAC<br>GGGUUGGAAACCAUAUGAUUGUAAAGGAAAGU
Ltr-S-Q493/G496/Q498-6	GGAUUUAGAGUACCCCAAAAAUGAAGGGGACUAAAAC<br>UGGGUUGGAAACCAUAUGAUUGUAAAGGAAAG
Ltr-S-Q493/G496/Q498-7	GGAUUUAGAGUACCCCAAAAAUGAAGGGGACUAAAAC<br>GUGGGUUGGAAACCAUAUGAUUGUAAAGGAAA
Ltr-S-Q493/G496/Q498-8	GGAUUUAGAGUACCCCAAAAAUGAAGGGGACUAAAAC<br>AGUGGGUUGGAAACCAUAUGAUUGUAAAGGAA
Ltr-S-R493/S496/R498-1	GGAUUUAGAGUACCCCAAAAAUGAAGGGGACUAAAAC<br>CGGAAACUAUAUGAUCGUAAAGGAAAGUAACA
Ltr-S-R493/S496/R498-2	GGAUUUAGAGUACCCCAAAAAUGAAGGGGACUAAAAC<br>UCGGAAACUAUAUGAUCGUAAAGGAAAGUAAC
Ltr-S-R493/S496/R498-3	GGAUUUAGAGUACCCCAAAAAUGAAGGGGACUAAAAC<br>GUCGGAAACUAUAUGAUCGUAAAGGAAAGUAA
Ltr-S-R493/S496/R498-4	GGAUUUAGAGUACCCCAAAAAUGAAGGGGACUAAAAC<br>GGUCGGAAACUAUAUGAUCGUAAAGGAAAGUA
Ltr-S-R493/S496/R498-5	GGAUUUAGAGUACCCCAAAAAUGAAGGGGACUAAAAC<br>GGGUCGGAAACUAUAUGAUCGUAAAGGAAAGU
Ltr-S-R493/S496/R498-6	GGAUUUAGAGUACCCCAAAAAUGAAGGGGACUAAAAC<br>UGGGUCGGAAACUAUAUGAUCGUAAAGGAAAG
Ltr-S-R493/S496/R498-7	GGAUUUAGAGUACCCCAAAAAUGAAGGGGACUAAAAC<br>GUGGGUCGGAAACUAUAUGAUCGUAAAGGAAA
Ltr-S-R493/S496/R498-8	GGAUUUAGAGUACCCCAAAAAUGAAGGGGACUAAAAC<br>AGUGGGUCGGAAACUAUAUGAUCGUAAAGGAA

Supplementary Table 4. IVT-tgRNA sequences used in this study.

Name	Sequence
SARS-CoV-N	AUGUCUGAUAAUGGACCCCAAAAUCAGCGAAAUGCACCCCGCAUUACGUUUGGUGGACCCUCAGAUU<br>CAACUGGCAGUAACCAGAAUGGAGAACGCAGUGGGGCGCGAUCAAAACAACGUCGGCCCCAAGGUUU<br>ACCCAAUAAUACUGCGUCUUGGUUCACCGCUCUCACUCAACAUGGCAAGGAAGACCUUAAAUUCCCU<br>CGAGGACAAGGCGUUCCAAUUAACACCAAUAGCAGUCCAGAUGACCAAAUUGGCUACUACCGAAGAG<br>CUACCAGACGAAUUCGUGGUGGUGACGGUAAAAUGAAAGAUCUCAGUCCAAGAUGGUAUUUCUACUA<br>CCUAGGAACUGGGCCAGAAGCUGGACUUCCCUAUGGUGCUAACAAAGACGGCAUCAUAUGGGUUGCA<br>ACUGAGGGAGCCUUGAAUACACCAAAAGAUCACAUUGGCACCCGCAAUCCUGCUAACAAUGCUGCAA<br>UCGUGCUACAACUUCCUCAAGGAACAACAUUGCCAAAAGGCUUCUACGCAGAAGGGAGCAGAGGCGG<br>CAGUCAAGCCUCUUCUCGUUCCUCAUCACGUAGUCGCAACAGUUCAAGAAAUUCAACUCCAGGCAGC<br>AGUAGGGGAACUUCUCCUGCUAGAAUGGCUGGCAAUGGCGGUGAUGCUGCUCUUGCUUUGCUGCUGC<br>UUGACAGAUUGAACCAGCUUGAGAGCAAAAUGUCUGGUAAAGGCCAACAACAACAAGGCCAAACUGU<br>CACUAAGAAAUCUGCUGCUGAGGCUUCUAAGAAGCCUCGGCAAAAACGUACUGCCACUAAAGCAUAC<br>AAUGUAACACAAGCUUUCGGCAGACGUGGUCCAGAACAAACCCAAGGAAAUUUUGGGGACCAGGAAC<br>UAAUCAGACAAGGAACUGAUUACAAACAUUGGCCGCAAAUUGCACAAUUUGCCCCCAGCGCUUCAGC<br>GUUCUUCGGAAUGUCGCGCAUUGGCAUGGAAGUCACACCUUCGGGAACGUGGUUGACCUACACAGGU<br>GCCAUCAAAUUGGAUGACAAAGAUCCAAAUUUCAAAGAUCAAGUCAUUUUGCUGAAUAAGCAUAUG

	ACGCAUACAAAACAUUCCCACCAACAGAGCCUAAAAAGGACAAAAAGAAGAAGGCUGAUGAAACUCA<br>AGCCUUACCGCAGAGACAGAAGAAACAGCAAACUGUGACUCUUCUUCCUGCUGCAGAUUUGGAUGAU<br>UUCUCCAAACAAUUGCAACAAUCCAUGAGCAGUGCUGACUCAACUCAGGCCUAA
CoV-S_1404-1523	gggUUCAACUGAAAUCUAUCAGGCCGGUAGCACACCUUGUAAUGGUGUUGAAGGUUUUAAUUGUUAC<br>UUUCCUUUACAAUCAUAUGGUUUCCAACCCACUAAUGGUGUUGGUUACCAACCAUA
CoV-S_E484K/N501Y_1404_1523	gggUUCAACUGAAAUCUAUCAGGCCGGUAGCACACCUUGUAAUGGUGUUAAAGGUUUUAAUUGUUAC<br>UUUCCUUUACAAUCAUAUGGUUUCCAACCCACUUAUGGUGUUGGUUACCAACCAUA
CoV-S_L452_1284-1403_r	gggUUUUACAGGCUGCGUUAUAGCUUGGAAUUCUAACAAUCUUGAUUCUAAGGUUGGUGGUAAUUAU<br>AAUUACCUGUAUAGAUUGUUUAGGAAGUCUAAUCUCAAACCUUUUGAGAGAGAUAU
CoV-S_R452_1284-1403_r	gggUUUUACAGGCUGCGUUAUAGCUUGGAAUUCUAACAAUCUUGAUUCUAAGGUUGGUGGUAAUUAU<br>AAUUACCGGUAUAGAUUGUUUAGGAAGUCUAAUCUCAAACCUUUUGAGAGAGAUAU
CoV-S_Omicron_1404-1523	gggUUCAACUGAAAUCUAUCAGGCCGGUAACAAACCUUGUAAUGGUGUUGCAGGUUUUAAUUGUUAC<br>UUUCCUUUACGAUCAUAUAGUUUCCGACCCACUUAUGGUGUUGGUCACCAACCAUA

Supplementary Table 5. DNA primers used in this study.

Name	Sequence
crRNA-T7P-f	GGATCCTAATACGACTCACTATA
Ltr-S-E484-1-r	TCAGGCCGGTAGCACACCTTGTAATGGTGTTG<br>GTTTTAGTCCCCTTCATTTTTGGGGTACTCTAAATCC TATAGTGAGTCGTATTAGGATCC
Ltr-S-E484-2-r	CAGGCCGGTAGCACACCTTGTAATGGTGTTGA<br>GTTTTAGTCCCCTTCATTTTTGGGGTACTCTAAATCC TATAGTGAGTCGTATTAGGATCC
Ltr-S-E484-3-r	AGGCCGGTAGCACACCTTGTAATGGTGTTGAA<br>GTTTTAGTCCCCTTCATTTTTGGGGTACTCTAAATCC TATAGTGAGTCGTATTAGGATCC
Ltr-S-E484-4-r	GGCCGGTAGCACACCTTGTAATGGTGTTGAAG<br>GTTTTAGTCCCCTTCATTTTTGGGGTACTCTAAATCC TATAGTGAGTCGTATTAGGATCC
Ltr-S-E484-5-r	GCCGGTAGCACACCTTGTAATGGTGTTGAAGG<br>GTTTTAGTCCCCTTCATTTTTGGGGTACTCTAAATCC TATAGTGAGTCGTATTAGGATCC
Ltr-S-E484-6-r	CCGGTAGCACACCTTGTAATGGTGTTGAAGGT<br>GTTTTAGTCCCCTTCATTTTTGGGGTACTCTAAATCC TATAGTGAGTCGTATTAGGATCC
Ltr-S-E484-7-r	CGGTAGCACACCTTGTAATGGTGTTGAAGGTT<br>GTTTTAGTCCCCTTCATTTTTGGGGTACTCTAAATCC TATAGTGAGTCGTATTAGGATCC
Ltr-S-E484-8-r	GGTAGCACACCTTGTAATGGTGTTGAAGGTTT<br>GTTTTAGTCCCCTTCATTTTTGGGGTACTCTAAATCC TATAGTGAGTCGTATTAGGATCC
Ltr-S-E484-9-r	GTAGCACACCTTGTAATGGTGTTGAAGGTTTT<br>GTTTTAGTCCCCTTCATTTTTGGGGTACTCTAAATCC TATAGTGAGTCGTATTAGGATCC
Ltr-S-E484-10-r	TAGCACACCTTGTAATGGTGTTGAAGGTTTTA<br>GTTTTAGTCCCCTTCATTTTTGGGGTACTCTAAATCC TATAGTGAGTCGTATTAGGATCC
Ltr-S-K484-1-r	TCAGGCCGGTAGCACACCTTGTAATGGTGTTA<br>GTTTTAGTCCCCTTCATTTTTGGGGTACTCTAAATCC TATAGTGAGTCGTATTAGGATCC
Ltr-S-K484-2-r	CAGGCCGGTAGCACACCTTGTAATGGTGTTAA<br>GTTTTAGTCCCCTTCATTTTTGGGGTACTCTAAATCC TATAGTGAGTCGTATTAGGATCC
Ltr-S-K484-3-r	AGGCCGGTAGCACACCTTGTAATGGTGTTAAA<br>GTTTTAGTCCCCTTCATTTTTGGGGTACTCTAAATCC TATAGTGAGTCGTATTAGGATCC
Ltr-S-K484-4-r	GGCCGGTAGCACACCTTGTAATGGTGTTAAAG<br>GTTTTAGTCCCCTTCATTTTTGGGGTACTCTAAATCC TATAGTGAGTCGTATTAGGATCC
Ltr-S-K484-5-r	GCCGGTAGCACACCTTGTAATGGTGTTAAAGG<br>GTTTTAGTCCCCTTCATTTTTGGGGTACTCTAAATCC TATAGTGAGTCGTATTAGGATCC
Ltr-S-K484-6-r	CCGGTAGCACACCTTGTAATGGTGTTAAAGGT<br>GTTTTAGTCCCCTTCATTTTTGGGGTACTCTAAATCC TATAGTGAGTCGTATTAGGATCC
Ltr-S-K484-7-r	CGGTAGCACACCTTGTAATGGTGTTAAAGGTT<br>GTTTTAGTCCCCTTCATTTTTGGGGTACTCTAAATCC TATAGTGAGTCGTATTAGGATCC
Ltr-S-K484-8-r	GGTAGCACACCTTGTAATGGTGTTAAAGGTTT<br>GTTTTAGTCCCCTTCATTTTTGGGGTACTCTAAATCC TATAGTGAGTCGTATTAGGATCC

Ltr-S-K484-9-r	GTAGCACACCTTGTAATGGTGTTAAAGGTTTT<br>GTTTTAGTCCCCTTCATTTTTGGGGTACTCTAAATCC TATAGTGAGTCGTATTAGGATCC
Ltr-S-K484-10-r	TAGCACACCTTGTAATGGTGTTAAAGGTTTTA<br>GTTTTAGTCCCCTTCATTTTTGGGGTACTCTAAATCC TATAGTGAGTCGTATTAGGATCC
Ltr-S-N501-1-r	TCCTTTACAATCATATGGTTTCCAACCCACTA<br>GTTTTAGTCCCCTTCATTTTTGGGGTACTCTAAATCC TATAGTGAGTCGTATTAGGATCC
Ltr-S-N501-2-r	CCTTTACAATCATATGGTTTCCAACCCACTAA<br>GTTTTAGTCCCCTTCATTTTTGGGGTACTCTAAATCC TATAGTGAGTCGTATTAGGATCC
Ltr-S-N501-3-r	CTTTACAATCATATGGTTTCCAACCCACTAAT<br>GTTTTAGTCCCCTTCATTTTTGGGGTACTCTAAATCC TATAGTGAGTCGTATTAGGATCC
Ltr-S-N501-4-r	TTTACAATCATATGGTTTCCAACCCACTAATG<br>GTTTTAGTCCCCTTCATTTTTGGGGTACTCTAAATCC TATAGTGAGTCGTATTAGGATCC
Ltr-S-N501-5-r	TTACAATCATATGGTTTCCAACCCACTAATGG<br>GTTTTAGTCCCCTTCATTTTTGGGGTACTCTAAATCC TATAGTGAGTCGTATTAGGATCC
Ltr-S-N501-6-r	TACAATCATATGGTTTCCAACCCACTAATGGT<br>GTTTTAGTCCCCTTCATTTTTGGGGTACTCTAAATCC TATAGTGAGTCGTATTAGGATCC
Ltr-S-N501-7-r	ACAATCATATGGTTTCCAACCCACTAATGGTG<br>GTTTTAGTCCCCTTCATTTTTGGGGTACTCTAAATCC TATAGTGAGTCGTATTAGGATCC
Ltr-S-N501-8-r	CAATCATATGGTTTCCAACCCACTAATGGTGT<br>GTTTTAGTCCCCTTCATTTTTGGGGTACTCTAAATCC TATAGTGAGTCGTATTAGGATCC
Ltr-S-N501-9-r	AATCATATGGTTTCCAACCCACTAATGGTGTT<br>GTTTTAGTCCCCTTCATTTTTGGGGTACTCTAAATCC TATAGTGAGTCGTATTAGGATCC
Ltr-S-N501-10-r	ATCATATGGTTTCCAACCCACTAATGGTGTTG<br>GTTTTAGTCCCCTTCATTTTTGGGGTACTCTAAATCC TATAGTGAGTCGTATTAGGATCC
Ltr-S-Y501-1-r	TCCTTTACAATCATATGGTTTCCAACCCACTT<br>GTTTTAGTCCCCTTCATTTTTGGGGTACTCTAAATCC TATAGTGAGTCGTATTAGGATCC
Ltr-S-Y501-2-r	CCTTTACAATCATATGGTTTCCAACCCACTTA<br>GTTTTAGTCCCCTTCATTTTTGGGGTACTCTAAATCC TATAGTGAGTCGTATTAGGATCC
Ltr-S-Y501-3-r	CTTTACAATCATATGGTTTCCAACCCACTTAT<br>GTTTTAGTCCCCTTCATTTTTGGGGTACTCTAAATCC TATAGTGAGTCGTATTAGGATCC
Ltr-S-Y501-4-r	TTTACAATCATATGGTTTCCAACCCACTTATG<br>GTTTTAGTCCCCTTCATTTTTGGGGTACTCTAAATCC TATAGTGAGTCGTATTAGGATCC
Ltr-S-Y501-5-r	TTACAATCATATGGTTTCCAACCCACTTATGG<br>GTTTTAGTCCCCTTCATTTTTGGGGTACTCTAAATCC TATAGTGAGTCGTATTAGGATCC
Ltr-S-Y501-6-r	TACAATCATATGGTTTCCAACCCACTTATGGT<br>GTTTTAGTCCCCTTCATTTTTGGGGTACTCTAAATCC TATAGTGAGTCGTATTAGGATCC
Ltr-S-Y501-7-r	ACAATCATATGGTTTCCAACCCACTTATGGTG<br>GTTTTAGTCCCCTTCATTTTTGGGGTACTCTAAATCC TATAGTGAGTCGTATTAGGATCC
Ltr-S-Y501-8-r	CAATCATATGGTTTCCAACCCACTTATGGTGT<br>GTTTTAGTCCCCTTCATTTTTGGGGTACTCTAAATCC TATAGTGAGTCGTATTAGGATCC
Ltr-S-Y501-9-r	AATCATATGGTTTCCAACCCACTTATGGTGTT<br>GTTTTAGTCCCCTTCATTTTTGGGGTACTCTAAATCC TATAGTGAGTCGTATTAGGATCC
Ltr-S-Y501-10-r	ATCATATGGTTTCCAACCCACTTATGGTGTTG<br>GTTTTAGTCCCCTTCATTTTTGGGGTACTCTAAATCC TATAGTGAGTCGTATTAGGATCC
Ltr-S-L452-1-r	GATTCTAAGGTTGGTGGTAATTATAATTACCT<br>GTTTTAGTCCCCTTCATTTTTGGGGTACTCTAAATCC TATAGTGAGTCGTATTAGGATCC
Ltr-S-L452-2-r	ATTCTAAGGTTGGTGGTAATTATAATTACCTG<br>GTTTTAGTCCCCTTCATTTTTGGGGTACTCTAAATCC TATAGTGAGTCGTATTAGGATCC
Ltr-S-L452-3-r	TTCTAAGGTTGGTGGTAATTATAATTACCTGT<br>GTTTTAGTCCCCTTCATTTTTGGGGTACTCTAAATCC TATAGTGAGTCGTATTAGGATCC
Ltr-S-L452-4-r	TCTAAGGTTGGTGGTAATTATAATTACCTGTA<br>GTTTTAGTCCCCTTCATTTTTGGGGTACTCTAAATCC TATAGTGAGTCGTATTAGGATCC
Ltr-S-L452-5-r	CTAAGGTTGGTGGTAATTATAATTACCTGTAT<br>GTTTTAGTCCCCTTCATTTTTGGGGTACTCTAAATCC TATAGTGAGTCGTATTAGGATCC
Ltr-S-L452-6-r	TAAGGTTGGTGGTAATTATAATTACCTGTATA<br>GTTTTAGTCCCCTTCATTTTTGGGGTACTCTAAATCC TATAGTGAGTCGTATTAGGATCC
Ltr-S-L452-7-r	AAGGTTGGTGGTAATTATAATTACCTGTATAG<br>GTTTTAGTCCCCTTCATTTTTGGGGTACTCTAAATCC TATAGTGAGTCGTATTAGGATCC
Ltr-S-L452-8-r	AGGTTGGTGGTAATTATAATTACCTGTATAGA<br>GTTTTAGTCCCCTTCATTTTTGGGGTACTCTAAATCC TATAGTGAGTCGTATTAGGATCC
Ltr-S-L452-9-r	GGTTGGTGGTAATTATAATTACCTGTATAGAT<br>GTTTTAGTCCCCTTCATTTTTGGGGTACTCTAAATCC TATAGTGAGTCGTATTAGGATCC
Ltr-S-L452-10-r	GTTGGTGGTAATTATAATTACCTGTATAGATT<br>GTTTTAGTCCCCTTCATTTTTGGGGTACTCTAAATCC TATAGTGAGTCGTATTAGGATCC
Ltr-S-R452-1-r	GATTCTAAGGTTGGTGGTAATTATAATTACCG<br>GTTTTAGTCCCCTTCATTTTTGGGGTACTCTAAATCC TATAGTGAGTCGTATTAGGATCC
Ltr-S-R452-2-r	ATTCTAAGGTTGGTGGTAATTATAATTACCGG<br>GTTTTAGTCCCCTTCATTTTTGGGGTACTCTAAATCC TATAGTGAGTCGTATTAGGATCC
Ltr-S-R452-3-r	TTCTAAGGTTGGTGGTAATTATAATTACCGGT<br>GTTTTAGTCCCCTTCATTTTTGGGGTACTCTAAATCC TATAGTGAGTCGTATTAGGATCC

Ltr-S-R452-4-r	TCTAAGGTTGGTGGTAATTATAATTACCGGTA<br>GTTTTAGTCCCCTTCATTTTTGGGGTACTCTAAATCC TATAGTGAGTCGTATTAGGATCC
Ltr-S-R452-5-r	CTAAGGTTGGTGGTAATTATAATTACCGGTAT<br>GTTTTAGTCCCCTTCATTTTTGGGGTACTCTAAATCC TATAGTGAGTCGTATTAGGATCC
Ltr-S-R452-6-r	TAAGGTTGGTGGTAATTATAATTACCGGTATA<br>GTTTTAGTCCCCTTCATTTTTGGGGTACTCTAAATCC TATAGTGAGTCGTATTAGGATCC
Ltr-S-R452-7-r	AAGGTTGGTGGTAATTATAATTACCGGTATAG<br>GTTTTAGTCCCCTTCATTTTTGGGGTACTCTAAATCC TATAGTGAGTCGTATTAGGATCC
Ltr-S-R452-8-r	AGGTTGGTGGTAATTATAATTACCGGTATAGA<br>GTTTTAGTCCCCTTCATTTTTGGGGTACTCTAAATCC TATAGTGAGTCGTATTAGGATCC
Ltr-S-R452-9-r	GGTTGGTGGTAATTATAATTACCGGTATAGAT<br>GTTTTAGTCCCCTTCATTTTTGGGGTACTCTAAATCC TATAGTGAGTCGTATTAGGATCC
Ltr-S-R452-10-r	GTTGGTGGTAATTATAATTACCGGTATAGATT<br>GTTTTAGTCCCCTTCATTTTTGGGGTACTCTAAATCC TATAGTGAGTCGTATTAGGATCC
Ltr-S-Q493/G496/Q498-1-r	tgttactttcctttacaatcatatggtttcca<br>GTTTTAGTCCCCTTCATTTTTGGGGTACTCTAAATCC TATAGTGAGTCGTATTAGGATCC
Ltr-S-Q493/G496/Q498-2-r	gttactttcctttacaatcatatggtttccaa<br>GTTTTAGTCCCCTTCATTTTTGGGGTACTCTAAATCC TATAGTGAGTCGTATTAGGATCC
Ltr-S-Q493/G496/Q498-3-r	ttactttcctttacaatcatatggtttccaac<br>GTTTTAGTCCCCTTCATTTTTGGGGTACTCTAAATCC TATAGTGAGTCGTATTAGGATCC
Ltr-S-Q493/G496/Q498-4-r	tactttcctttacaatcatatggtttccaacc<br>GTTTTAGTCCCCTTCATTTTTGGGGTACTCTAAATCC TATAGTGAGTCGTATTAGGATCC
Ltr-S-Q493/G496/Q498-5-r	actttcctttacaatcatatggtttccaaccc<br>GTTTTAGTCCCCTTCATTTTTGGGGTACTCTAAATCC TATAGTGAGTCGTATTAGGATCC
Ltr-S-Q493/G496/Q498-6-r	ctttcctttacaatcatatggtttccaaccca<br>GTTTTAGTCCCCTTCATTTTTGGGGTACTCTAAATCC TATAGTGAGTCGTATTAGGATCC
Ltr-S-Q493/G496/Q498-7-r	tttcctttacaatcatatggtttccaacccac<br>GTTTTAGTCCCCTTCATTTTTGGGGTACTCTAAATCC TATAGTGAGTCGTATTAGGATCC
Ltr-S-Q493/G496/Q498-8-r	ttcctttacaatcatatggtttccaacccact<br>GTTTTAGTCCCCTTCATTTTTGGGGTACTCTAAATCC TATAGTGAGTCGTATTAGGATCC
Ltr-S-R493/S496/R498-1-r	TGTTACTTTCCTTTACGATCATATAGTTTCCG<br>GTTTTAGTCCCCTTCATTTTTGGGGTACTCTAAATCC TATAGTGAGTCGTATTAGGATCC
Ltr-S-R493/S496/R498-2-r	GTTACTTTCCTTTACGATCATATAGTTTCCGA<br>GTTTTAGTCCCCTTCATTTTTGGGGTACTCTAAATCC TATAGTGAGTCGTATTAGGATCC
Ltr-S-R493/S496/R498-3-r	TTACTTTCCTTTACGATCATATAGTTTCCGAC<br>GTTTTAGTCCCCTTCATTTTTGGGGTACTCTAAATCC TATAGTGAGTCGTATTAGGATCC
Ltr-S-R493/S496/R498-4-r	TACTTTCCTTTACGATCATATAGTTTCCGACC<br>GTTTTAGTCCCCTTCATTTTTGGGGTACTCTAAATCC TATAGTGAGTCGTATTAGGATCC
Ltr-S-R493/S496/R498-5-r	ACTTTCCTTTACGATCATATAGTTTCCGACCC<br>GTTTTAGTCCCCTTCATTTTTGGGGTACTCTAAATCC TATAGTGAGTCGTATTAGGATCC
Ltr-S-R493/S496/R498-6-r	CTTTCCTTTACGATCATATAGTTTCCGACCCA<br>GTTTTAGTCCCCTTCATTTTTGGGGTACTCTAAATCC TATAGTGAGTCGTATTAGGATCC
Ltr-S-R493/S496/R498-7-r	TTTCCTTTACGATCATATAGTTTCCGACCCAC<br>GTTTTAGTCCCCTTCATTTTTGGGGTACTCTAAATCC TATAGTGAGTCGTATTAGGATCC
Ltr-S-R493/S496/R498-8-r	TTCCTTTACGATCATATAGTTTCCGACCCACT<br>GTTTTAGTCCCCTTCATTTTTGGGGTACTCTAAATCC TATAGTGAGTCGTATTAGGATCC
CoV-N-FL-PCR-f	GGATCCTAATACGACTCACTATAGGATGTCTGATAATGGACCCCAAAATCAGC
CoV-N-FL-PCR-r	TTAGGCCTGAGTTGAGTCAGCACTG
CoV-S_1404-1523_r	tatggttggtaaccaacaccattagtgggttggaaaccatatgattgtaaaggaaagtaaca<br>attaaaaccttcaacaccattacaaggtgtgctaccggcctgatagatttcagttgaa ccc<br>TATAGTGAGTCGTATTAGGATCC
CoV-S(E484K/N501Y) 1404-1523_r	tatggttggtaaccaacaccataagtgggttggaaaccatatgattgtaaaggaaagtaaca<br>attaaaacctttaacaccattacaaggtgtgctaccggcctgatagatttcagttgaa ccc<br>TATAGTGAGTCGTATTAGGATCC

CoV-S_ L452_1284-1403_r	ATATCTCTCTCAAAAGGTTTGAGATTAGACTTCCTAAACAATCTATACAGGTAATTATAATT<br>ACCACCAACCTTAGAATCAAGATTGTTAGAATTCCAAGCTATAACGCAGCCTGTAAAA ccc<br>TATAGTGAGTCGTATTAGGATCC
CoV-S_ R452_1284-1403_r	ATATCTCTCTCAAAAGGTTTGAGATTAGACTTCCTAAACAATCTATACCGGTAATTATAATT<br>ACCACCAACCTTAGAATCAAGATTGTTAGAATTCCAAGCTATAACGCAGCCTGTAAAA ccc<br>TATAGTGAGTCGTATTAGGATCC
CoV-S_Omicron_1404-1523_r	TATGGTTGGTGACCAACACCATAAGTGGGTCGGAAACTATATGATCGTAAAGGAAAGTAACA<br>ATTAAAACCTGCAACACCATTACAAGGTTTGTTACCGGCCTGATAGATTTCAGTTGAA ccc<br>TATAGTGAGTCGTATTAGGATCC

Supplementary Table 6. Information of SARS-CoV-2 RNA from patients.

ID	Ct value	Conc. (copies/μL)	Lineage	Mutations
1	ND	ND	-	-
2	ND	ND	-	-
3	ND	ND	-	-
4	ND	ND	-	-
5	ND	ND	-	-
6	ND	ND	-	-
7	ND	ND	-	-
8	ND	ND	-	-
9	ND	ND	-	-
10	ND	ND	-	-
11	22.339	58,051	B.1.1.214	L5F, D614G
12	23.465	28,421	B.1.1.214	V62I, D614G, S813I
13	23.106	35,683	B.1.1.214	D614G, Q675H
14	23.840	22,409	B.1.1.214	D614G, Q675H
15	18.756	563,350	B.1.1.214	L54F, D614G, P681L
16	27.788	1,831	B.1.1.284	D614G, Q677H
17	27.279	2,529	B.1.1.284	M153T, D614G
18	19.950	264,207	B.1.1.284	T29I, M153T, D614G
19	21.871	78,095	B.1.1.284	M153T, D614G
20	25.154	9,735	B.1.1.284	M153T, D614G, S640F
21	24.067	19,400	B.1.1.7	H69_V70del, Y145del, N501Y, A570D, D614G, P681H, T716I, S982A, D1118H
22	26.217	4,960	B.1.1.7	H69_V70del, Y145del, N501Y, A570D, D614G, P681H, T716I, S982A, D1118H
23	24.879	11,592	B.1.1.7	H69_V70del, Y145del, N501Y, A570D, D614G, P681H, T716I, S982A, D1118H
24	24.866	11,684	B.1.1.7	L5F, H69_V70del, Y145del, N501Y, A570D, D614G, P681H, T716I, S982A, D1118H
25	23.750	23,718	B.1.1.7	H69_V70del, Y145del, N501Y, A570D, D614G, P681H, T716I, S982A, D1118H
26	21.063	130,359	B.1.1.7	H69_V70del, Y145del, N501Y, A570D, D614G, P681H, T716I, S982A, D1118H, L1200F
27	24.078	19,269	B.1.1.7	H69_V70del, Y145del, N501Y, A570D, D614G, P681H, T716I, S982A, D1118H
28	24.987	10,826	B.1.1.7	H69_V70del, Y145del, N501Y, A570D, D614G, P681H, T716I, S982A, D1118H
29	22.036	70,359	B.1.1.7	H69_V70del, Y145del, N501Y, A570D, D614G, P681H, T716I, S982A, D1118H

30	24.642	13,472	B.1.1.7	H69_V70del, Y145del, N501Y, A570D, D614G, P681H, T716I, S982A, D1118H
31	24.055	19,543	AY.29	T19R, E156_R158delinsG, L452R, T478K, D614G, P681R, D950N
32	20.702	163,914	AY.29	T19R, E156_R158delinsG, L452R, T478K, D614G, P681R, D950N
33	18.419	697,680	AY.29	T19R, T95I, G142D, E156_R158delinsG, L452R, T478K, D614G, P681R, D950N, N1119T
34	17.781	1,045,807	AY.29	T19R, T95I, G142D, E156_R158delinsG, L452R, T478K, D614G, P681R, D950N
35	21.936	74,954	AY.29	T19R, T95I, G142D, E156_R158delinsG, L452R, T478K, D614G, P681R, D950N
36	23.297	31,619	AY.29	T19R, T95I, G142D, E156_R158delinsG, L452R, T478K, D614G, P681R, D950N
37	23.450	28,693	AY.29	T19R, T95I, G142D, E156_R158delinsG, L452R, T478K, D614G, P681R, D950N
38	18.999	482,792	AY.29	T19R, T95I, G142D, E156_R158delinsG, L452R, T478K, D614G, P681R, D950N
39	27.408	2,330	AY.29	T19R, T95I, G142D, E156_R158delinsG, A243S, L452R, T478K, D614G, P681R, D950N
40	14.830	6,794,308	AY.29	T19R, T95I, G142D, E156_R158delinsG, L452R, T478K, D614G, P681R, D950N
41	24.592	13,906	R.1	W152L, E484K, D614G, G769V
42	21.881	77,638	R.1	W152L, E484K, D614G, G769V
43	21.779	82,809	R.1	W152L, E484K, D614G, G769V
44	19.627	324,156	R.1	W152L, E484K, D614G, G769V
45	30.503	327	R.1	W152L, E484K, D614G, G769V
46	21.997	72,093	R.1	W152L, E484K, D614G, G769V
47	21.093	127,926	R.1	W152L, E484K, D614G, G769V
48	20.873	147,088	R.1	W152L, E484K, D614G, G769V
49	26.751	3,535	R.1	W152L, E484K, D614G, G769V, L1203F
50	25.612	7,283	R.1	R78M, W152L, E484K, D614G, G769V
51	27.709	1,926	BA.1	A67V, H69_V70del, T95I, G142_Y145delinsD, N211_L212delinsI, R214_D215insEPE, G339D, R346K, S371L, S373P, S375F, K417N, N440K, G446S, S477N, T478K, E484A, Q493R, G496S, Q498R, N501Y, Y505H, T547K, D614G, H655Y, N679K, P681H, N764K, D796Y, N856K, Q954H, N969K, L981F
52	25.433	8,157	BA.1	A67V, H69_V70del, T95I, G142_Y145delinsD, N211_L212delinsI, R214_D215insEPE, G339D, S371L, S373P, S375F, K417N, N440K, G446S, S477N, T478K, E484A, Q493R, G496S, Q498R, N501Y, Y505H, T547K, D614G, H655Y, N679K, P681H, N764K, D796Y, N856K, Q954H, N969K, L981F
53	26.283	4,756	BA.1	A67V, H69_V70del, T95I, G142_Y145delinsD, N211_L212delinsI, R214_D215insEPE, G339D, R346K, S371L, S373P, S375F, K417N, N440K, G446S, S477N, T478K, E484A, Q493R, G496S, Q498R, N501Y, Y505H, T547K, D614G, H655Y, N679K, P681H, N764K, D796Y, N856K, Q954H, N969K, L981F
54	26.684	3,690	BA.1	A67V, H69_V70del, T95I, G142_Y145delinsD, N211_L212delinsI, R214_D215insEPE, G339D,

				R346K, S371L, S373P, S375F, K417N, N440K, G446S, S477N, T478K, E484A, Q493R, G496S, Q498R, N501Y, Y505H, T547K, D614G, H655Y, N679K, P681H, N764K, D796Y, N856K, Q954H, N969K, L981F
55	24.651	13,391	BA.1	A67V, H69_V70del, T95I, G142_Y145delinsD, N211_L212delinsI, R214_D215insEPE, G339D, S371L, S373P, S375F, K417N, N440K, G446S, S477N, T478K, E484A, Q493R, G496S, Q498R, N501Y, Y505H, T547K, D614G, H655Y, N679K, P681H, N764K, D796Y, N856K, Q954H, N969K, L981F
56	22.520	51,743	BA.1	A67V, H69_V70del, T95I, G142_Y145delinsD, N211_L212delinsI, R214_D215insEPE, G339D, R346K, S371L, S373P, S375F, K417N, N440K, G446S, S477N, T478K, E484A, Q493R, G496S, Q498R, N501Y, Y505H, T547K, D614G, H655Y, N679K, P681H, N764K, D796Y, N856K, Q954H, N969K, L981F
57	26.990	3,038	BA.1	A67V, H69_V70del, T95I, G142_Y145delinsD, N211_L212delinsI, R214_D215insEPE, G339D, R346K, S371L, S373P, S375F, K417N, N440K, G446S, S477N, T478K, E484A, Q493R, G496S, Q498R, N501Y, Y505H, T547K, D614G, H655Y, N679K, P681H, N764K, D796Y, N856K, Q954H, N969K, L981F
58	25.257	9,122	BA.1	A67V, H69_V70del, T95I, G142_Y145delinsD, N211_L212delinsI, R214_D215insEPE, G339D, R346K, S371L, S373P, S375F, K417N, N440K, G446S, S477N, T478K, E484A, Q493R, G496S, Q498R, N501Y, Y505H, T547K, D614G, H655Y, N679K, P681H, N764K, D796Y, N856K, Q954H, N969K, L981F
59	27.680	1,961	BA.1	A67V, H69_V70del, T95I, G142_Y145delinsD, N211_L212delinsI, R214_D215insEPE, G339D, R346K, S371L, S373P, S375F, K417N, N440K, G446S, S477N, T478K, E484A, Q493R, G496S, Q498R, N501Y, Y505H, T547K, D614G, H655Y, N679K, P681H, N764K, D796Y, N856K, Q954H, N969K, L981F
60	27.101	2,832	BA.1	A67V, H69_V70del, T95I, G142_Y145delinsD, N211_L212delinsI, R214_D215insEPE, G339D, R346K, S371L, S373P, S375F, K417N, N440K, G446S, S477N, T478K, E484A, Q493R, G496S, Q498R, N501Y, Y505H, T547K, D614G, H655Y, N679K, P681H, N764K, D796Y, N856K, Q954H, N969K, L981F

Supplementary Table 7. Assay cost of opn-SATORI.

Material	Cost (US$)
CD	<0.01
Buffer	0.09

Mineral oil	0.01
FQ reporter	0.87
Alexa647	0.61
MyOne T1	0.29
NHS-dPEG4-biotin	0.01
LtrCas13a	~0.02
crRNA	0.07
Total	1.97
