# Supplementary material for: Automated amplification-free digital RNA detection platform for rapid and sensitive SARS-CoV-2 diagnosis
Source: Commun Biol. 2022 May 26;5:473. doi: 10.1038/s42003-022-03433-6 (PMC9132978; doi:10.1038/s42003-022-03433-6)
Supplement: Supplementary file 2 — Description of Additional Supplementary Files [file 42003_2022_3433_MOESM2_ESM.pdf]

Description of Additional Supplementary Files

File name: Supplementary Data 1
Description: All source data used for generating graphs and charts in main and supplementary figures.
